# Supplementary figures and images for: The effects of 0.9% saline versus Plasma-Lyte 148 on renal function as assessed by creatinine concentration in patients undergoing major surgery: A single-centre double-blinded cluster crossover trial
Source: PLoS One. 2021 May 19;16(5):e0251718. doi: 10.1371/journal.pone.0251718 (PMC8133498; doi:10.1371/journal.pone.0251718)

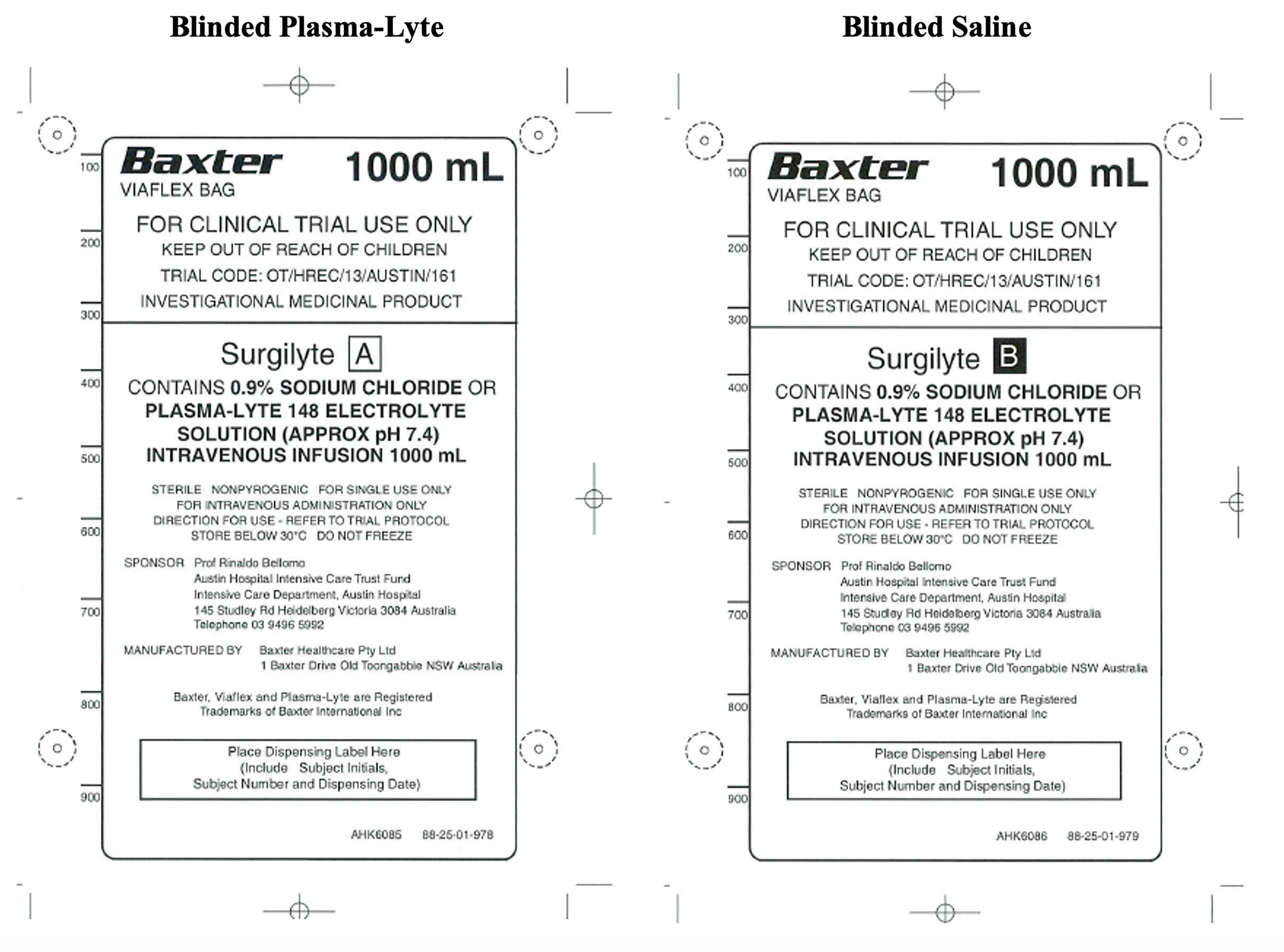

Supplement: S1 Fig — (JPG) [file pone.0251718.s002.jpg]
