# Supplementary material for: The effects of 0.9% saline versus Plasma-Lyte 148 on renal function as assessed by creatinine concentration in patients undergoing major surgery: A single-centre double-blinded cluster crossover trial
Source: PLoS One. 2021 May 19;16(5):e0251718. doi: 10.1371/journal.pone.0251718 (PMC8133498; doi:10.1371/journal.pone.0251718)
Supplement: S1 Table — (DOCX) [file pone.0251718.s003.docx]

**S1 Table.** Physiochemical differences of the types of fluids administered compared to human plasma.

|  | **Plasma** | **Saline** | **Plasma-Lyte 148** |
| --- | --- | --- | --- |
| **Sodium** (mmol L^-1^) | 136 - 145 | 154 | 140 |
| **Potassium** (mmol L^-1^) | 3.5 - 5.0 | 0 | 5 |
| **Magnesium** (mmol L^-1^) | 0.8 - 1.0 | 0 | 1.5 |
| **Calcium** (mmol L^-1^) | 2.2 - 2.6 | 0 | 0 |
| **Chloride** (mmol L^-1^) | 98 - 106 | 154 | 98 |
| **Acetate** (mmol L^-1^) | 0 | 0 | 27 |
| **Gluconate** (mmol L^-1^) | 0 | 0 | 23 |
| **Lactate** (mmol L^-1^) | 0.5-2.0 | 0 | 0 |
| **Octanoate** (mmol L^-1^) | 0 | 0 | 0 |
| **eSID** (mEq L^-1^) | 42 | 0 | 50 |
| **Theoretical osmolarity** (mOsm L^-1^) | 291 | 308 | 295 |
| **Actual or measured *osmolality** (mOsm kg^-1^ H_2_O) | 287 | 286 | 271 |
| **pH** | 7.35 - 7.45 | 4.5 – 7 | 4 - 8 |

* Freezing point depression

Plasma-Lyte 148 manufactured by Baxter Healthcare, Toongabie, NSW, Australia

Hartmann’s solution manufactured by Baxter Healthcare, Toongabie, NSW, Australia
