# Supplementary material for: The effects of 0.9% saline versus Plasma-Lyte 148 on renal function as assessed by creatinine concentration in patients undergoing major surgery: A single-centre double-blinded cluster crossover trial
Source: PLoS One. 2021 May 19;16(5):e0251718. doi: 10.1371/journal.pone.0251718 (PMC8133498; doi:10.1371/journal.pone.0251718)
Supplement: S2 Table — (DOCX) [file pone.0251718.s004.docx]

**S2 Table.** Differences in perioperative variables in patients with measured and unmeasured renal function tests.

|  | **Unmeasured**  **Renal function**  **(n=113)** | | | **Measured**  **renal function**  **(n=947)** | | | **All patients**  **(n=1060)** | | |
| --- | --- | --- | --- | --- | --- | --- | --- | --- | --- |
|  | **Saline** | **PlasmaLyte** | **p value** | **Saline** | **PlasmaLyte** | **p value** | **Measured**  **renal function** | **Unmeasured**  **renal function** | **p value** |
| **Preoperative characteristics** | | | | | | | | | |
| No of patients | 63 (10.5%) | 50 (10.9%) | - | 539 (56.9%) | 408 (43.1%) | - | 947 | 113 |  |
| Age, years | 50.4 (19.6) | 41.8 (18.8) | 0.020 | 61.2 (17.7) | 61.7 (17.3) | 0.686 | 64 (50:5) | 44 (32:63) | <0.001 |
| Male gender | 36 (57.1%) | 29 (58%) | >0.999 | 356 (66.0.%) | 236 (57.9) | 0.010 | 527 (55.6) | 65 (57.5) | 0.764 |
| Charlson Comorbidity Index | 0 (0:0) | 0 (0:0) | 0.751 | 1 (0:3) | 1 (0:2) | 0.267 | 1 (0-3) | 0 (0:0) | <0.001 |
| Serum Creatinine, μmol L^-1^ | 72 (62:87) | 71 (64:78) | 0.801 | 78 (65:96) | 77 (65:96) | 0.734 | 77 (65:96) | 72 (64:82) | 0.003 |
| eGFR, mL min^-1^ 1.73m^-2^ | 91 (78:91) | 91 (85.5:91) | 0.381 | 84 (63:91) | 82.5 (61:91) | 0.476 | 83 (63:91) | 91 (83:91) | <0.001 |
| Serum chloride, μmol L^-1^ | 101 (99:102) | 101 (100:102.8) | 0.186 | 100 (98:103) | 103 (98:103) | 0.848 | 100 (98:103) | 101 (100:102) | 0.144 |
| Serum bicarbonate, μmol L^-1^ | 27 (25:28) | 26 (25:28) | 0.215 | 26 (24:28) | 26 (24:28) | 0.076 | 26 (24:28) | 27 (25:28) | 0.070 |
| Anaemia (WHO definition) | 8 | 2 | 0.196 | 179 (33.2%) | 127 (31.1%) | 0.527 | 298 (32.6) | 13 (15.1) | <0.001 |
| Haemoglobin, g L^-1^ | 136 (127:145) | 144 (133:153) | 0.094 | 133 (120:146) | 134 (119:145) | 0.756 | 133 (120:145) | 139 (130:148) | 0.003 |
| **Surgery type** | | | | | | | | | |
| Emergency surgery | 19 (30.2%) | 12 (24%) | 0.528 | 238 (44.2%) | 149 (35.5%) | 0.012 | 387 (40.9) | 31 (27.4) | 0.006 |
| Cardiothoracic | 1 (1.6%) | 3 (6%) | 0.565 | 88 (16.3%) | 72 (17.6%) | 0.810 | 160 (16.9%) | 4 (3.5%) | <0.001 |
| Major abdominal | 0 (0%) | 1 (2%) |  | 82 (15.2%) | 67 (16.4%) |  | 149 (15.7%) | 1 (0.9%) |  |
| Major orthopaedic | 11 (17.5%) | 9 (18%) |  | 130 (24.1%) | 86 (21.1%) |  | 216 (22.8%) | 20 (17.8%) |  |
| Vascular | 2 (3.2%) | 2 (4%) |  | 47 (8.8%) | 33 (8.1%) |  | 80 (8.5%) | 4 (3.5%) |  |
| Other surgery | 49 (77.7%) | 35 (70%) |  | 192 (35.6%) | 150 (36.8%) |  | 342 (36.1%) | 84 ((74.3%) |  |
| **Intra- and postoperative variables** | | | | | | | | | |
| Duration of surgery, mins | 149 (130:179) | 155.0 (134.3:195.3) | 0.185 | 210 (160:300) | 205 (155.5:293.5) | 0.663 | 207 (158:295) | 153 (131:183) | <0.001 |
| Intraoperative fluid, mL | 1000 (1000:2000) | 1000 (1000:2000) | 0.586 | 2000 (1000:2000) | 2000 (1000:2000) | 0.236 | 2000 (1000:2000) | 1000 (1000:2000) | <0.001 |
| Number of complications per  patient | 0 (0:0) | 0 (0:0) | 0.416 | 1 (0:4) | 1 (0:4) | 0.809 | 1 (0:4) | 0 (0:0) | <0.001 |
| Length of stay, days | 1 (1:2) | 1 (1:3) | 0.234 | 7 (4:11) | 6 (4:12) | 0.042 | 6 (4:12) | 1 (1:2) | <0.001 |
