# Supplementary material for: The effects of 0.9% saline versus Plasma-Lyte 148 on renal function as assessed by creatinine concentration in patients undergoing major surgery: A single-centre double-blinded cluster crossover trial
Source: PLoS One. 2021 May 19;16(5):e0251718. doi: 10.1371/journal.pone.0251718 (PMC8133498; doi:10.1371/journal.pone.0251718)
Supplement: S1 Protocol — (PDF) [file pone.0251718.s006.pdf]

CLINICAL TRIAL PROTOCOL

Saline (0.9%) vs. **P**lasma-**L**yte® 148 Fluid **I**ntervention **T**rial in Major Surgery Patients (The SPLIT- Major Surgery study)

A single-centred double-blind pilot trial investigating the safety and efficacy of using 0.9% saline or Plasmalyte® 148 as fluid therapy in adult patients undergoing major surgery

Protocol Version: Version 3
Date 1st Oct 2014

GENERAL INFORMATION 4
1.1 Chief Investigator 4
1.2 Principle Investigators 4
1.3 Participating centre 4
1.4 Coordinating department 4
STUDY SYNOPSIS 5
Title: 5
Short Title: 5
Design: 5
Study Centers: 5
Hospital: 5
Study question: 5
Study Objectives: 5
Primary outcome: 5
Secondary Outcomes: 5
ABBREVIATIONS 8
LAY SUMMARY 8
1.5 Fluid therapy in patients receiving major surgery 8
1.6 Saline (0.9%) vs. Plasmalyte 8
1.7 Study design 8
1.8 Study importance 9
1.9 Hospitals involved 9
1.10 Number of participants 9
FLOW DIAGRAM 10
RESEARCH QUESTION 11
BACKGROUND AND RATIONALE 11
FEASIBILITY AT AUSTIN HOSPITAL 15
1.11 Summary 19
OBJECTIVES 19
1.12 General 19
1.13 Inclusion criteria 20
1.14 Exclusion criteria 20
1.15 Baseline data 20
1.16 Study treatments 20
1.17 Treatment allocation 21
TREATMENT SCHEME 22
Outcome measures 22
1.17.1 General 22
1.17.2 Primary outcome measure 23
1.17.3 Secondary outcome measures 23
ETHICS 23
1.18 Guiding principles 23
1.19 Ethical and legal issues in this study 23
1.20 Confidentiality of patient data 24
DATA MANAGEMENT 25
1.21 Data collection methods 25

1.22 Data management 25
1.23 Protocol deviation 25
SAFETY CONSIDERATIONS 26
1.24 Adverse Events and Other Safety Aspects 26
1.25 Definition of Adverse Events 26
1.26 Reporting of Severe Adverse Events 28
1.27 Criteria for Causal Relationship to the Trial Fluid 29
1.28 Criteria for Defining the Severity of an Adverse Event 29
STATISTICAL CONSIDERATIONS 30
1.29 Power calculations and sample size 30
1.30 Analysis plan 30
1.31 Sub-groups 31
1.32 Randomization process 31
1.33 2 Blinding 31
STUDY BUDGET 32
1.34 Detailed budget 33
1.35 Dispensing and storage costs 33
STUDY ADMINISTRATION STRUCTURE 34
1.36 Department of Anaesthesia & Intensive Care responsibilities 34
1.37 Data management responsibilities 34
1.38 Management committee responsibilities 34
FUNDING 34
PUBLICATIONS 34
REFERENCES 35

GENERAL INFORMATION

1.1 Chief Investigator

Name:	Dr Laurence Weinberg
Title:	Staff Specialist in Anaesthesia, Austin Hospital.
Address:	145 Studley Rd, Heidelberg, VIC, 3084, Australia
Contact Number:	+61-3-9496-5000
Fax number:	+61-3-9459-6421
Email:	Laurence.Weinberg@austin.org.au

1.2 Principle Investigators

Name:	Dr Glenn Eastwood
Title:	Research Officer, Austin Hospital.
Address:	145 Studley Rd, Heidelberg, VIC, 3084, Australia
Contact Number:	+61-3-9496-5000
Fax number:	+61-3-9459-6421
Email:	Glenn.Eastwood@austin.org.au

Name:	Mr Kent Garrett
Title:	Director of Pharmacy, Austin Hospital.
Address:	145 Studley Rd, Heidelberg, VIC, 3084, Australia
Contact Number:	+61-3-9496-5000
Fax number:	+61-3-9459-6421
Email:	Kent.Garrett@austin.org.au

Name:	Professor Rinaldo Bellomo
Title:	Head of Research, Department of Intensive Care, Austin Hospital.
Address:	145 Studley Rd, Heidelberg, VIC, 3084, Australia
Contact Number:	+61-3-9496-5000
Fax number:	+61-3-9459-6421
Email:	Rinaldo.Bellomo@austin.org.au

1.3 Participating centre

Austin Hospital

1.4 Coordinating department

Department of Anaesthesia
Austin Hospital
Heidelberg, Victoria
Australia

STUDY SYNOPSIS

**Title:**	A single-centred randomised double-blind pilot trial investigating the safety and efficacy of using 0.9% saline or Plasmalyte® 148 as fluid therapy in adult patients undergoing major surgery
**Short Title:**	0.9% **S**aline vs. **P**lasma-**L**yte® 148 for fluid **I**ntervention **T**rial in Major Surgery Patients<br>**(The SPLIT- Major Surgery study)**
**Design:**	**Phase 4, single center blinded interventional study**<br>Patients will be randomly assigned, in alternating 6 weeks blocks to use either blinded Saline (0.9%) or Plasmalyte solution. There will be a three week wash-in period and a three-week wash-out period between each fluid intervention<br><br>The total study duration will be 4 months
**Study Centers:**	1
**Hospital:**	Austin Hospital
**Study question:**	Of the commonly used perioperative crystalloid solutions: Saline (0.9%) and Plasmalyte, which solution has the most favourable effect on kidney injury, serum electrolyte levels, and complications in patients undergoing major surgery
**Study Objectives:**	To determine the safety and efficacy of using Saline (0.9%) or Plasmalyte solution as fluid therapy in adult patients undergoing major surgery
**Primary outcome:**	Acute kidney injury or failure (RIFLE-criteria)
**Secondary Outcomes:**	Serum creatinine<br>Chloride levels<br>Acid base disturbances<br>Post-operative complications<br>Requirements for ICU<br>Duration of ICU stay<br>Hospital length of stay<br>In-hospital mortality
**Inclusion criteria:**	Adult patients > 18 years<br>Elective or emergency surgery<br>Requiring at least one overnight stay
**Exclusion criterion:**	<18 years in age<br>Expected hospital stay < 1 day<br>Raised intracranial pressure<br>Liver transplantation<br>Renal transplantation

**Number of planned Subjects:**	**2000**
**Investigational product:**	Baxter Healthcare (Pty) will supply all Trial Fluids<br><br>**Name:** Plasmalyte® 148<br>Dose: At the discretion of all treating clinicians as per standard care<br><br>**Name:** Normal saline (0.9%)<br>Dose: At the discretion of all treating clinicians as per standard care
**Safety considerations:**	Whilst this study is an interventional study most of its features that are more akin to an observational study.<br><br>**NB: Specifically, the study involves no departure from standard care and does not involve the collection of any data that are not already being collected for clinical or quality assurance purposes.**<br><br>**All other fluid e.g. blood products, colloids, dextrose, bicarbonate etc., will still remain at the discretion of the treating clinicians.**
**Statistical Methods:**	A complete description of the statistical analyses will be specified in a statistical analyses plan, finalised prior to completion of the study.<br><br>All analyses will be conducted on an intention-to-treat basis.<br><br>Primary analyses: will be unadjusted analyses in which binary outcomes will be compared using relative risks with 95% confidence intervals and chi square tests and continuous outcomes will be compared with the use of mean differences and un-paired T-tests assuming that normality assumptions are meet.<br><br>If normality assumptions data transformation will be attempted, such as a logarithm transformation, and if this fails to proceed to a Mann-Whitney rank based test.<br><br>Adjusted analyses: will be performed using Poisson regression for binary outcomes and linear regression for continuous outcomes. Baseline covariates will include age, gender, elective vs.

	emergency surgery, surgical specialty of admission, type of operation, and baseline serum creatinine level. Survival times will be compared using log-rank tests and presented as Kaplan-Meier curves.
**Subgroups:**	Patients undergoing abdominal surgery<br>Patients undergoing vascular surgery<br>Patients undergoing thoracic surgery<br>Patients undergoing other surgeries (orthopaedics, urology etc.)<br>Patients undergoing emergency surgery

ABBREVIATIONS

**NS**	Normal saline (0.9%)
**IV**	Intravenous
**CPB**	Cardiopulmonary bypass
**ECGs**	Electrocardiographs
**ICU**	Intensive Care Unit
**APACHE**	Acute Physiology and Chronic Health Evaluation
**AE**	Adverse event
**CRF**	Case Report Form
**SAE**	Serious adverse event

LAY SUMMARY

1.5 Fluid therapy in patients receiving major surgery

The administration of intravenous (IV) crystalloid fluid (also known as fluid therapy) is a ubiquitous intervention in patients undergoing surgery. Worldwide, the most commonly used crystalloid fluids available for patients undergoing major surgery include Saline (0.9%) or the balanced fluid solutions - Hartmann's and Plasmalyte. Choice of fluid therapy is based frequently dependent on geography[1], and choice of fluid amongst anaesthetists at Austin Hospital is similar to worldwide practices.

1.6 Saline (0.9%) vs. Plasmalyte

Saline has been used in clinical practice for fluid therapy since the late 1800s. While it is the most commonly used IV fluid in the world[1] recent data raise the possibility that it might increase the risk of developing kidney damage in acutely unwell patients compared to fluids with lower concentrations of chloride such as Plasmalyte[2.] While this increased risk of kidney damage with the use of saline is plausible[3,] current data are insufficient to recommend clinical practice change[4] and data from an interventional trial are urgently needed. However, the design of such a Trial requires sufficient pilot data to establish feasibility, safety power calculations and define an optimal study protocol.

1.7 Study design

This study aims use an interventional design to provide high quality pilot data in a rapid time frame to address this important question and to help decide whether a pivotal randomized controlled trial is justified. Thus, all patients undergoing surgery at Austin Hospital will be randomly assigned to receive either Saline (0.9%) or the balanced crystalloid solution Plasmalyte as the primary crystalloid fluid in a blinded fashion during two study periods.

The study will be conducted over a 4-month period allowing two interventional periods of 6 weeks each, with a three week wash-in and a three week wash-out period for each group. Blinded study fluid will be used for all crystalloid therapy in all participants. Austin Hospital staff specialists in anaesthesia agree that on the basis of current evidence, Saline (0.9%) and Plasmalyte are equally acceptable for crystalloid fluid therapy in every situation requiring major surgery with the exclusion of surgery for liver transplantation, renal transplantation and patients with raised intracranial pressure, where specific hospital perioperative fluid protocols are used in these settings.

Both Saline (0.9%) and Plasmalyte will be available for open-label administration in the rare situations, where, in the opinion of the treating anaesthetist or clinician, there is a clinical indication for one fluid or the other. This may occur, for example, in the setting of severe acidosis where the higher chloride content of saline may make this fluid problematic because it may increase the severity of such acidosis[5].

All adult patients who receive blinded or open-label fluid during one of the two-month study blocks will be analysed. The primary outcome will be the proportion of patients with kidney injury or failure based on established criteria[6]. Secondary outcomes will include delta creatinine (the difference between baseline and peak creatinine), serum electrolyte levels, bicarbonate level, requirement for renal replacement therapy, complications and in-hospital mortality.

All of the data required for this study are collected routinely as part of standard clinical care and/or quality assurance activities[7].

1.8 Study importance

The study will establish the pilot feasibility, safety and preliminary efficacy evidence base for the design of a large interventional trial to inform clinicians looking after major surgery patients as to whether Saline (0.9%) or Plasmalyte or solution is the preferred crystalloid fluid in this setting.

1.9 Hospitals involved

Austin Hospital

1.10 Number of participants

Given the current theatre workload at Austin Hospital, approximately 1000 participants will be recruited in each group. A total of 2000 participants are expected.

FLOW DIAGRAM

\

0.9% **S**aline vs. **P**lasma **L**yte® 148 for **F**luid **I**ntervention **T**rial in **Major Surgery** Patients
(The SPLIT- Major Surgery study)

⇩

HYPOTHESIS

Of the commonly used perioperative crystalloid solutions, Plasmalyte solution has more favourable effect on kidney injury, serum electrolyte levels, and complications in patients undergoing major surgery compared to Saline (0.9%)

⇩

Inclusion Criteria

- ✓ Adult patients > 18 years
- ✓ Elective or emergency surgery
- ✓ Requiring at least one overnight stay

Exclusion criteria

- <18 years in age
- Expected hospital stay < 1 day
- Raised intracranial pressure
- Liver transplantation
- Renal transplantation

⇩

INTERVENTIONAL STUDY WITH BLINDED PLASMALYTE OR SALINE SOLUTIONS (order depending on randomisation)

Wash-in Trial Fluid A (3 weeks)
STUDY Trial Fluid A (6 weeks)
Wash-out Trial Fluid A (3 weeks)

Wash-in Trial Fluid B (3 weeks)
STUDY Trial Fluid B (6 weeks)
Wash-out Trial Fluid B (3 weeks)

⇩

Primary outcome

Acute kidney injury or failure (RIFLE-criteria)

Secondary outcomes

Serum creatinine
Chloride levels
Acid base disturbances
Post-operative complications
Requirements for ICU
Duration of ICU stay
Hospital length of stay
In-hospital mortality

RESEARCH QUESTION

Of the two commonly used perioperative crystalloid solutions: Saline (0.9%), and Plasmalyte, which solution has the most favourable effect on kidney injury, serum electrolyte levels, and complications in adult patients undergoing major surgery in a university teaching hospital?

BACKGROUND AND RATIONALE

For all patients undergoing major surgery, two important and fundamental goals in fluid intervention include the maintenance of intravascular volume to ensure optimal organ perfusion and function, and the avoidance of metabolic and electrolyte disturbances that can compromise organ function.

Worldwide, the most commonly used crystalloid fluids available for all patients undergoing surgery include:

1. Balanced solutions, commonly Plasmalyte solution or Hartmann's solution
2. Saline (0.9%)

The electrolyte composition of some of these commonly used crystalloid fluids are summarised in Table 1. All these solutions have similar sodium concentrations (130-150 mmol/L) and may contain physiological concentrations of potassium (Hartmann's and Plasmalyte solutions). There are however some major differences in anion composition. All three solutions above have chloride as a major anionic constituent, the balance in Hartmann's and Plasmalyte solutions being made up of lactate and acetate respectively. Both lactate and acetate are ultimately metabolised by the liver to bicarbonate, thus producing a near ideal physiological solution. Because their electrolyte composition is similar to plasma, they are frequently called "balanced" crystalloid solutions.

Pre-clinical and early clinical data suggest the saline may give rise to adverse effects including immune dysfunction[8,] gastrointestinal dysfunction[9] and decreased renal cortical perfusion and renal blood flow[10]. There is strong expert opinion that 'normal saline' or saline(0.9%) is neither "normal" nor "physiological"[11]. In fact, it has approximately 1.5 times more chloride than normal plasma and its use can lead to hyperchloraemic acidosis[3]. Although hyperchloraemic acidosis may be a benign phenomenon[12,13], recent data raise the possibility that the use of saline for fluid resuscitation in adult ICU patients may lead to an increased risk of developing acute kidney injury compared to resuscitation fluids with a lower concentration of chloride such as Plasmalyte[2,14].

In a single centre prospective open-label sequential period pilot study of 1533 critically ill patients, the implementation of a chloride-restrictive strategy that included avoiding the use of normal saline was associated with a significant decrease in the incidence of acute kidney injury and use of renal replacement

therapy[2]. Similarly, a large retrospective study of adults undergoing major open abdominal surgery suggested that, compared to saline, the use of Plasmalyte was associated with a decreased risk of major complications including the development of renal failure requiring dialysis[14].

Plasmalyte is a crystalloid that more closely resembles plasma with a more physiological chloride concentration and pH (Table 1). Yet, there is a lack of well-designed prospective studies comparing Plasmalyte to other crystalloid solutions. The direct measurement of the "unmeasured" anions acetate and gluconate has recently been investigated by Davies et al[15]. Thirty adult patients were systematically randomized to 1:1 to CPB prime with either bicarbonate-balanced fluid (24 mmol/L bicarbonate) or Plasmalyte. Acetate concentrations (normal 0.04 - 0.07 mmol/L) became markedly elevated at 3 minutes after CPB commencement, where the Plasmalyte group (median 3.69, range [2.46 - 8.55]) exceeded the bicarbonate group (0.16 [0.02 - 3.49], P <0.0005). Immediately before CPB separation, levels had declined but the differential pattern remained apparent. Normal circulating acetate concentrations were not restored until 4 hours post separation from CPB.

Similarly gluconate concentration profiles and inter-group differences were seen, with a slower decay immediately before CPB separation. IL-6 increased across CPB, peaking at 4 hours post separation from CPB, with no clear difference between groups. To date this is the only study to demonstrate that acetate containing prime solutions result in supraphysiological plasma concentrations of acetate. The use of acetate-free prime fluid in CPB significantly reduced but did not eliminate large acetate surges in cardiac surgical patients.

Acetate surges in the vasculature may not be benign. In renal replacement therapy, the pro-inflammatory, vasodilatory, myocardial depressant and hypoxaemia promoting properties of acetate[9-15] has led to its removal from contemporary renal replacement fluids. Acetate remains an integral component of commonly used CPB pump prime solutions. Prior to the Davies paper[15], little was known of the acetate concentration profile and consequent physiological impact during cardiac surgery incorporating exposure to acetate-based fluid. The situation concerning gluconate is equally unclear, despite its widespread use.

It therefore remains unresolved from whether the demonstrated supra-physiological concentrations of acetate or gluconate can cause harm. The answer cannot be found in small non-randomised, incompletely matched cohort study, particularly since substitution of bicarbonate in the circuit prime in the Davies study[15] failed to eliminate exposure to supra-physiological concentrations of acetate and gluconate. However, there is already unequivocal evidence of acetate toxicity in contexts other than CPB. A number of studies have documented hypoxia and hypotension when patients with end stage renal disease were dialysed against solutions containing acetate[17-19]. There is also evidence of cytokine release, carbohydrate

intolerance, disturbances of fatty acid synthesis, reduction of cytosolic redox potential, intracellular accumulation of phosphate, pyrophosphate, phosphorylated intermediates and calcium, and deposition of intra-mitochondrial calcium and magnesium pyrophosphate[18].

Acetate has also been implicated in direct myocardial toxicity. Patients with chronic renal failure receiving acetate-free haemodiafiltration achieved better stroke volumes, demonstrated a lesser reduction in peripheral resistance and recorded smaller troponin increases than patients receiving conventional acetate-based dialysis[22]. In an isolated perfused rat heart model, exposure of myocardial tissue to acetate concentrations as low as 5 mmol/L resulted in impaired fatty acid oxidation and decreased ATP turnover[21]. Finally, Plasmalyte 148, although promoted as a resuscitation solution, performed poorly in a haemorrhagic shock model. Traverso et al compared four resuscitation crystalloids[23], and found that Plasmalyte was associated with a lower survival and a late rise in plasma L-lactate concentrations as compared with normal saline and Ringer's lactate solutions.

Acetate-based haemodialysis in Australia and elsewhere has been abandoned, even in supplementary concentrations although it continues to be an integral component of 'balanced' resuscitation fluids, CPB priming solutions and total parenteral nutrition. During CPB, adverse effects triggered by acetate exposure would be difficult to detect amongst the vigorous metabolic and host defence responses to surgery[24], hypothermia, and non-pulsatile blood flow. Although there is no proven detrimental effect, the concentrations reported by Davies suggest a need for further investigations into the safety of acetate containing fluids. At present acetate still remains an integral component of commonly used cardiopulmonary pump prime solutions, and to date there is no clinical evidence to suggest that supra-physiological concentrations of the acetate anion could cause harm. Recently, plasmalyte has been extensively used in patients undergoing major abdominal surgery and recent evidence suggests that it may exert renoprotecive compared to normal saline[14].

Hartmann's solution has been widely recommended in the medical literature as it causes less acidosis than saline[25-19]. However, Hartmann's solution is not completely balanced when compared with plasma; with a higher chloride and lower pH. Some authors have questioned whether the lactate in Hartmann's solution may aggravate the lactic acidosis and/or alter the sensitivity and specificity of plasma lactate as a prognostic marker, including after liver resection[30]. In the context of liver resection surgery, liver dysfunction, which can be pre-existing and/or as a result of a reduction in liver capacity after liver resection, can impair the metabolism of lactate found in Hartmann's solution. This may result in iatrogenic hyperlactaemia, which may confound the clinical picture if plasma lactate is being used as a marker of critical illness. Plasma lactate has been demonstrated to be an important prognostic marker after liver resection, correlating well with an increased risk of complications and death[30,31]. Because of these reasons some authors have

suggested avoiding Hartmann's solution during liver resection despite a lack of evidence[32].

While current data are of insufficient quality to recommend practice change[4], establishing the relative efficacy and safety of using Saline (0.9%) compared to Plasmalyte in acutely ill adults is now an important research priority.

No large-scale interventional trial has compared saline (0.9%) to a lower chloride crystalloid solution. The balanced crystalloid solutions are the logical comparators to saline (0.9%) for a large scale crystalloid trial. There may be additional clinical advantages of Plasmalyte over Hartmann's solution because, unlike other commercially available low-chloride crystalloid solutions, Plasmalyte is compatible with blood products preserved in citrate-based anticoagulation solutions because it does not contain calcium[3] and also because it contains a more physiological concentration of chloride than any other crystalloid solution. In addition Plasmalyte does not contain the anion lactate, which is an important marker of illness severity as outlined above.

Large scale fluid trials using conventional randomised controlled trials are feasible; however, they are extremely expensive and time-consuming to conduct and can only be justified if initial work in the population of interest (major surgery patients) provides preliminary evidence of a possible effect. The recently completed CHEST study comparing saline (0.9%) to the colloid hydroxyethyl starch for fluid resuscitation in intensive care trial[33] in which the Austin Hospital was involved randomised 7000 patients and took 10 years to from inception to publication and cost approximately $5 million dollars to complete.

This proposal outlines a research approach that has the potential to provide key preliminary information and data for logistic feasibility, amounts of fluids used, safety data on the incidence of adverse events and preliminary outcome data for power calculations. Such information is crucial to the design of a subsequent pivotal trial.

Table 1. Concentration of ions in intravenous fluids*

Strong Ion	Plasma**	Plasmalyte	Saline(0.9%)	Hartmann's
Sodium (Na^+), meq/L	140 (135 to 145)	140	150	150
Chloride (Cl^-), meq/L	102 (98 to 108)	98	150	150
Potassium (K^+), meq/L	4.0 (3.5 to 5.0)	5	0	0
Calcium (Ca^{2+}), meq/L	2.4 (2.3 to 2.6)	0	0	0
Magnesium (Mg^{2+}), meq/L	2.0 (1.4 to 2.4)	3.0	0	0
Acetate, meq/L	0	27	0	0
Gluconate meq/L	0	23	0	0
Lactate, meq/L	1.0 (0.5 to 2.0)	0	0	0
Strong Ion Difference meq/L	44	49	0	0

*All fluids are manufactured by Baxter Healthcare, Toongabie, NSW.
**Median, Reference Range

FEASIBILITY AT AUSTIN HOSPITAL

The departments of anaesthesia and Intensive Care at Austin Hospital have completed 6 fluid intervention studies, and currently there is one fluid intervention study still underway in the setting of renal transplantation.

Fluid intervention in liver resection

Recently, the authors completed a multicentre randomized double-blind controlled multicentre study of Plasmalyte vs. Hartmann's solution in patients receiving liver resection[34]. Participants were randomized to Plasmalyte or Hartmann's solution for intraoperative fluid intervention. Primary outcome: base-excess immediately after surgery. Secondary outcomes: lactate levels, strong-ion-difference (SID), total weak acids, net-unmeasured-ions, changes in liver enzymes, perioperative complications and duration of hospital stay. Results: 60 participants were recruited from 4 tertiary-level hospitals. Both groups were matched according to baseline characteristics, extent of resection, and surgery duration. There were no differences in the volume of trial fluid used, perioperative fluid balance, or urine output. Plasmalyte was not inferior to Hartmann's for the primary endpoint. Plasmalyte base-excess was 0.8mmol/L greater (95%CI: -0.4 to 2.0;P=0.18). This was associated with hyperchloraemia (Difference 1.7mmol/L, 95%CI: 0.2 to 3.2mmol/L, p=0.03) and hyperlactaemia (Difference 0.8mmol/L, 95%CI: 0.2 to 1.3mmol/L;P=0.01) in the Hartmann's group. In the Hartmann's group 23 patients (77%) had a lactate above the reference range compared to 14 patients (47%) in the Plasmalyte group, P=0.02. Complications were more frequent in the Hartmann's Group (56% vs. 20%, 95%CI: 1.3 to 6.1;P=0.007). Median length of hospital stay: 5.9 days vs. 7.8 days (P=0.041) favouring Plasmalyte group.

Conclusion: For patients undergoing liver resection use of Plasmalyte solution resulted in improved acid base haemostasis, less hyperlactaemia, reduced perioperative complications and a shorter length of hospital stay compared to patients receiving Hartmann's solution.

Fluids and cognitive function in healthy volunteers
In another study, the authors tested the hypothesis that saline infusion would produce greater cognitive changes than Plasmalyte in healthy volunteers[35]. With Ethics Committee approval, we conducted a randomized, cross-over, blinded study of healthy adult volunteers. On separate days participants were randomized to 30 ml/kg over one hour of either saline or Plasmalyte. Plasma chemistry was tested on venous samples. As part of a battery of cognitive tests our primary end point was the reaction time index. We studied 25 participants. Plasma chloride was greater after saline, difference 5.4 mmol/L (95%CI: 4.1 to 6.6 mmol/L, P<0,001) associated with greater metabolic acidosis: base-excess 2.5 mmol/L more negative (95%CI: 1.9 to 3.0 mmol/L more negative, P <0.001). There were no important differences in reaction time index between the two arms of the study. After saline, the mean reaction time index was 411 (SD: 63) msec, and after Plasmalyte was 385 (SD: 55) msec: saline 9 msec slower (95 CI: 12 msec faster to 30 msec slower, P = 0.39). None of the other cognitive and mood tests differed.
We concluded that despite significant differences in plasma chemistry, reaction times after saline did not differ from reaction times after Plasmalyte. Further, other measures of cognition did not differ. This finding was contrary to our hypothesis. We cannot exclude differences with Hartmann's solution, however, cognitive differences associated with mild hyperchloremic metabolic acidosis seem unlikely.

Fluid intervention in cardiac surgery

The authors have successfully completed 4 fluid intervention studies in the setting of cardiac surgery.

Most recently, we examined the effect of pump prime on acidosis, strong-ion-difference and unmeasured ions during cardiopulmonary bypass in a randomized blinded clinical trial[36]. There are no studies comparing the mechanism of metabolic acidosis during cardiopulmonary bypass (CPB) using Hartmann's solution and Plasmalyte as pump primes, therefore we tested the hypothesis that the effects of these crystalloids on acidosis is a function of their individual strong ion differences (SID) and unmeasured anions. After Ethics approval we performed a randomised blinded study of 38 adult patients undergoing elective CABG or valve replacement requiring CPB. Both groups received a prime solution of 2000mL; one group with anions lactate and chloride (Hartmann's), the other with anions acetate, gluconate and chloride (Plasmalyte). Endpoints were standard base deficit, SID, total weak acids, and strong ion gap (SIG). Serum electrolytes and arterial blood gases were collected at 6 intervals: immediately prior to CPB (T0), then 2min (T1), 5min

(T2), 10min (T3), 30min (T4), and 60min (T5) post CPB. On delivery of pump prime both groups developed at metabolic acidosis – Plamsalyte® (base excess: 0.53mmol/L (T0) to -3.03mmol/L (T1), P=0.001, Hartmann's; base excess: 0.42mmol/L (T0) to -2.20mmol/L (T1), P=0.001. Lactate remained unchanged in the Plasmalyte group, however peaked with Hartmann's at 2min: 0.15mmol/L (T0) to 4.5mmol/L (T1), P=0.001, returning to baseline by 60minutes. There was significant hyperchloraemia with Hartmann's compared to Plasmalyte. The SID with Plasmalyte® increased from 37.6mEqu/L (T0) to 40.3mEqu/L (T1), P=0.001, remaining elevated at 60minutes (36.7mEqu/L). Conversely with Hartmann's, the SID decreased from 36.3mEqu/L (T0) to 31.2mEqu/L (T1) and remained decreased at T5 (33.3mEqu/L). The SIG increased significantly from T0 to T1 with Plasmalyte® (1.1mEqu/L to 11.9mEqu/L, P=0.001), but marginally with Hartmann's (-0.6mEqu/L to 2.0mEqu/L). We concluded that the mechanism of acidosis during CPB with Hartmann's solution was due to combination of iatrogenic hyperlactaemia and hyperchloraemia. In contrast the mechanism with Plasmalyte was a production of unmeasured anions, most likely acetate and gluconate.

Second, we previously studied acid-base changes during CPB with polygeline pump prime and defined and quantified the factors, which contribute to metabolic acidosis[27]. Using quantitative biophysical methods, we demonstrated that in patients receiving a pump prime rich in chloride and polygeline, the metabolic acidosis of CPB was mostly due to iatrogenic increases in serum chloride concentration and unmeasured strong anions.

Its development was partially attenuated by iatrogenic hypoalbuminaemia. Changes in lactate concentrations did not play a role in the development of metabolic acidosis in our patients.

Because the development of metabolic acidosis during cardiopulmonary bypass was well recognized but poorly understood, in another study we hypothesized that the delivery of pump prime fluids is primarily responsible for its development[28]. We studied acid-base changes induced by the establishment of CPB using two types of priming fluid (Haemaccel, a polygeline solution, and Ringer's Injection vs. Plasmalyteusing quantitative biophysical approach. Immediately on delivery of pump prime fluids, all patients developed a metabolic acidosis. The decrease in base excess was the same for both primes (4.60 vs. 4.37; not significant). However, the mechanism of metabolic acidosis was different. With the Haemaccel-Ringer's prime, the metabolic acidosis was hyperchloremic, whilst with Plasmalyte, the acidosis was induced by an increase in unmeasured anions, most probably acetate and gluconate, although these were not directly measured. The resolution of these two processes was different because the excretion of chloride was slower than that of the unmeasured anions. This study demonstrated that cardiopulmonary bypass–induced metabolic acidosis was iatrogenic in nature and derived from the effect of pump prime fluid on acid-base balance.

Finally, we tested the hypothesis that a cardiopulmonary bypass prime with lactate would be associated with less acidosis than a prime with only chloride anions because of differences in the measured strong-ion-difference. We randomised 20 patients to a 1500 ml bypass prime with either a chloride-only solution (Ringer's Injection) or a lactated solution (Hartmann's solution). We found that the chloride-only group had greater acidosis with lower base-excess and pH. Contrary to our hypothesis, however, the difference between the groups was not due to a difference in the measured strong-ion- difference. When the difference in standard base-excess between the groups was greatest, the difference in the measured strong-ion-difference was only very small. There was however, a difference in the net-unmeasured-ions (strong-ion-gap). We concluded that acid-base changes with cardiopulmonary bypass might differ with the prime but that the early differences between chloride-only and lactated primes appear not to be due to differences in the measured strong-ion-difference. We suggested that future studies examine other possible mechanisms including unmeasured ions.

Fluid intervention in renal transplantation

Currently the authors are half way through a randomised trial evaluating the use of either Saline (0.9%) or Plasmalyte solution in patients undergoing renal transplantation[37]. We hypothesised that the balanced crystalloid fluid Plasmalyte, will have more favourable effects on metabolic acidosis and early graft function compared to Saline (0.9%). Adult patients (age > 18 years) undergoing deceased donor renal transplantation (heart-beating or non heart-beating) are included with the primary endpoint being base deficit immediately post surgery in the Post Anaesthesia Care Unit and at 24 hours & 48 hrs postoperatively. A variety of secondary outcomes are being measured including potassium levels, strong-ion-difference, renal biomarkers, requirements for postoperative dialysis, adverse events and hospital stay. (days). We have completed recruitment of 20 patients with a total of 50 participants expected. There are no results at this early stage.

The department of anaesthesia and intensive care have an established research infrastructure and have participated in a number of large scale studies leading to a number of recent NEJM publications[38-42].

CHEST Study and SAFE Studies

Of note, the Austin Hospital contributed substantially to the 7000-patient RCT comparing saline (0.9%) to the colloid hydroxyethyl starch for fluid resuscitation in intensive care, which was recently published in the NEJM[33]. In addition, the ICU at Austin Hospital was a major recruiting centre for the multicenter, randomized, double-blind SAFE trial that compared the effect of fluid resuscitation with albumin or saline (0.9%) on mortality in a heterogeneous population of patients in the ICU[43]. This has been one of the most widely sited fluid intervention studies in the world.

1.11 Summary

The demographic, illness severity and outcome data that will account for the majority of data used in this study are already being collected. Existing data suggest that our study will have ample statistical power to establish whether using saline as the default resuscitation fluid increases the risk of acute kidney injury in adult major surgery patients compared to Plasmalyte. The statistical team at the Australian and New Zealand Intensive Care Research Centre, who will conduct the statistical analyses, are familiar with the data being used in the study.

Baxter Pty will provide blinded study fluids for this study. In short, this study has a very high chance of success. It should provide high quality pilot data for a subsequent pivotal, NHMRC-funded, double-blind randomized multicentre pragmatic interventional trial that will inform clinical practice around the world.

OBJECTIVES

The overall objective of this study is to establish the feasibility, safety and relative biochemical and physiological efficacy of using saline (0.9%) vs. Plasmalyte solution as the primary fluids in adult major surgery patients.

The choice of non-crystalloid fluid intervention such as colloids, or any other blood product e.g. red blood cells, platelets, cryoprecipitate, frozen plasma, bicarbonate, dextrose etc. will be entirely at the discretion of the all clinicians caring for the patients

Specifically, we aim to establish the effect of saline (0.9%) and Plasmalyte solution on:

1. The development of acute kidney injury in hospital
2. The development of biochemical and acid-base derangements
3. The development of postoperative complications
4. Requirements for renal replacement therapy
5. Requirements for mechanical ventilation
6. Hospital length of stay
7. Hospital mortality

STUDY DESIGN

1.12 General

This study is a single-centre pilot prospective pilot study comparing saline (0.9%) and Plasmalyte solution for all perioperative crystalloid fluid therapy in adult major surgery patients.

1.13 Inclusion criteria

- Patients aged ≥18 years admitted to the Austin Hospital who receive major surgery. For the purpose of the study, major surgery will be defined as any surgery requiring overnight post-operative hospital stay.

1.14 Exclusion criteria

- Patients who are <18 years in age
- Patients who are expected to be discharged from hospital on the day of surgery (day surgery cases)
- Raised intracranial pressure (these patients may specific fluid intervention strategies to prevent further increases in intracranial pressure)
- Patients undergoing liver transplantation (these patients require non-lactated crystalloid solutions as part of the Austin Hospital perioperative fluid intervention protocol.
- Patients undergoing renal transplantation, as these patients are currently part of a current randomised clinical trial.

1.15 Baseline data

The following baseline data will be collected:

- Age
- Gender
- Admission type (elective vs. emergency)
- Type of surgery (hepatobiliary, colorectal, upper GI, orthopaedic etc.)
- If admitted to ICU: Chronic APACHE co-morbidities (including long-term dialysis)
- If admitted to ICU: APACHE-III admission diagnosis[44]
- If admitted to ICU: Illness severity based on the on the APACHE-III risk of death score[44]
- Baseline creatinine.
- Baseline biochemistry

(Note: - all of these data are routinely collected for quality assurance purposes or as measured as part of usual clinical care)

1.16 Study treatments

This study is designed to compare two approaches to fluid therapy. It will compare use of Saline (0.9%) or Plasmalyte as the primary default crystalloid fluids.

Blinded study fluid will be provided by Baxter Pty in indistinguishable 1000ml bags. Open label fluids will be available for use in rare situations where the treating clinician believes that there is an indication for one fluid or the other. This may occur, for example, in the setting of severe acidosis where the higher chloride content of saline may make this fluid undesirable because it may increase the risk of more severe acidosis[5].

Anaesthetists and surgeons, and intensivsits, as well as all clinicians at Austin Hospital have indicated that they regard the study fluids as equally acceptable in essentially every clinical setting in which crystalloid fluid therapy is required. Irrespective of which study fluid the patient receives they will be analysed on an intention to treat basis.

The overall volume of study fluid and any open label study fluid administered as fluid boluses will be recorded daily.

1.17 Treatment allocation

Patients will be randomly assigned, in alternating 6 weeks blocks to use either blinded Saline (0.9%) or Plasmalyte solution. There will be a three week wash-in period and a three-week wash-out period between each fluid intervention.

For example, assume:

GROUP ONE: Saline (Blinded)

GROUP TWO: Plasmalyte (Blinded)

Three weeks prior to commencement, blinded Saline will be introduced into all theatres, ICU and surgical wards for a 3 week wash-in period. **GROUP ONE** subjects will then receive Saline (blinded) for 6 weeks. This will be followed by a 3-week wash-out period of Saline (blinded). Plasmalyte solution (blinded) will then be washed-in for 3 weeks. **GROUP TWO** subjects will then receive Plasmalyte solution (blinded) for a 6 week period. Plasmalyte solution (blinded) will be washed-out for 3 weeks and the study will stop.

Total study duration: 24 weeks

Any patients who remain in hospital throughout the transition period will continue to receive the blinded fluid that they were originally assigned to for all postoperative crytalloid interevention. Thus blinded fluid will be made available as the only crystalloid fluid in all surgical wards and in the intensive care unit. No controls will be placed on the use of colloidal fluid therapy, blood products (red blood cells, platelets, cryoprecipitate or fresh frozen plasma, or any other fluid non-crystalloid fluid intervention e.g. dextrose, bicarbonate.

TREATMENT SCHEME

Options will vary depending on final randomisation. Assume:

Group 1: Plasmalyte (Blinded)
Group 2: Saline (0.9%) (Blinded)

STUDY START

Wash-in with blinded Plasmalyte

3 weeks

GROUP 1

Plasmalyte Trial fluid (blinded) for 6 weeks then stop

Wash-out with blinded Plasmayte solution (3 weeks)

Wash-in with blinded Saline (0.9%) (3 weeks)

GROUP 2

Saline (0.9%) Trial fluid (blinded) for 6 weeks then stop

Wash-out with blinded Saline (0.9%) (3 weeks)

Wash-in with blinded Saline (3 weeks)

STUDY COMPLETED

Outcome measures

1.17.1 General

The outcome measures are aligned with those used in a recently published open-label sequential period study of chloride liberal vs. chloride-restrictive fluid administration study published in JAMA[2].

1.17.2 Primary outcome measure

The primary outcome measure will be the proportion of patients with either acute kidney injury or failure based on creatinine levels in accordance with RIFLE-criteria[12] during the index hospital admission.

1.17.3 Secondary outcome measures

Secondary outcomes will be

- the incidence of acute kidney injury based on creatinine according to risk, injury, failure, loss, end-stage (RIFLE) classification in the first 3 days hours after surgery,
- the increase between baseline and peak chloride levels in the first 3 days after surgery
- the development of metabolic acidosis (defined as a base deficit > 2 mEq/L or a bicarbonate level <20 mmol/L) in the first 3 days after surgery
- the development of acidemia defined as a pH <7.3 in the first 3 days after surgery
- Postoperative complications
- Need for and duration of renal replacement therapy,
- Proportion of patients requiring mechanical ventilation
- Need for ICU admission
- ICU and hospital length of stay, and
- ICU and in-hospital mortality

ETHICS

1.18 Guiding principles

This study is to be performed in accordance with World Medical Association Declaration of Helsinki: Ethical principles for medical research involving human subjects (WMA 2008), the International Ethical Guidelines for Biomedical Research Involving Human Subjects (CIOMS 2002) and the National Statement on Good Practice in Human Research

The research utilises information normally gathered during the course of the delivery of a currently recognised treatment and will maintain anonymity and privacy and can therefore be conducted in accordance with Australian Law and the National Statement.

1.19 Ethical and legal issues in this study

Patterns of practice in different hospitals are often idiosyncratic and unscientific[22]. Indeed, much of clinical medicine remains empirical and local medical opinion and supply of resources are often more important than science in determining how medical care is delivered[22]. Wide variations that characterise usual clinical practice often have no basis in science but may have important implications for patient outcomes. Where there are two

distinct approaches being employed in different institutions, we believe that there is an ethical imperative to conduct research to establish which approach is best.

This study utilises a novel design in order to assess the relative efficacy of three standard treatment approaches. Although this study is clearly an interventional study on the basis of conventional Guidelines, it has many features that are more akin to an observational study than an interventional one. Specifically, the study involves no departure from standard care and does not involve the collection of any data that are not already being collected for clinical and / or quality assurance purposes.

In this study, whether or not patients receive saline or Plasmalyte as default crystalloid fluid therapies will be determined, on the basis of whether they are cared for during a particular period. No controls will be placed on the use of colloidal fluid therapy, blood products (red blood cells, platelets, cryoprecipitate or fresh frozen plasma, or any other fluid non-crystalloid fluid intervention e.g. dextrose, bicarbonate.

All patients in this study will receive standard treatment except that the default therapy that they receive will be randomly allocated according to the 6-week block in which they are admitted to the study hospital.

At present, these treatments are already allocated in a quasi-random fashion based on which hospital patients attend or which doctors are involved in patient care. Attending doctors will have full discretion to use whichever study fluid they choose if a specific indication for one fluid or the other arises.

As a result of these factors, this study involves negligible risk.

The study design will provide high quality information about an important clinical question from a very large number of subjects in a short period of time. Given the low risk nature of the research, we will use a process of **'opt-out consent'** that involves the provision of information to patients and their next of kin and the opportunity to opt-out from the use of their data if they wish.

1.20 Confidentiality of patient data

Patients will be allocated a unique study number. Study data will be obtained from routinely collected quality assurance and clinical information. Data entered into the study database will be identified by the unique study number only. The enrolment log and the study data will be kept separately.

DATA MANAGEMENT

1.21 Data collection methods

The demographic, biochemical, and outcome data used in this study are already collected by the Austin Hospital for administrative and quality assurance purposes[7].

Any ICU data are similarly collected and already provided to the Australian and New Zealand Intensive Care Society Centre for Outcome and Resource Evaluation as part of a government-funded bi-national quality assurance programme.

The only data used in this study which are not routinely collected for quality assurance purposes are the daily volume of study fluid administered as fluid boluses and the data relating to renal outcomes. These data will be collected by the Principle investiogators or trained Research Co-ordinators.

1.22 Data management

De-identified data management will be performed by the Australian and New Zealand Intensive Care Research Centre (ANZIC-RC) located within the School of Epidemiology and Public Health of Monash University.

1.23 Protocol deviation

A protocol deviation is generally an unplanned excursion from the protocol that is not implemented or intended as a systematic change. The Chief investigator will be is responsible for ensuring the study is conducted in accordance with the procedures and evaluations described in this protocol and will protect the rights, safety, and welfare of all subjects. The Chief investigator will not implement any deviation from, or changes of, the protocol, unless it is necessary to eliminate an immediate hazard to trial subjects.

For the purposes of this protocol, any deviations requiring notification to the Research Ethics Committee will be defined as any participant who

- Entered into the study even though they did not satisfy the entry criteria.
- Developed exclusion criteria during the study and not withdrawn.

When a deviation from the protocol is identified for an individual subject, the chief investigator will assess the deviation and the possible impact to the safety and /or efficacy of the subject to determine subject continuation in the study. If a deviation impacts the safety of a subject, the Chief investigator will inform the Research Ethics Committee in accordance with normal Hospital processes.

SAFETY CONSIDERATIONS

1.24 Adverse Events and Other Safety Aspects

Safety will be assessed throughout the study. As part of normal hospital care for all patients undergoing surgery at Austin Hospital, a complete baseline profile of each subject will be established by the treating surgical unit through medical history, clinical laboratory values, vital signs, physical assessments, and ECGs. During the course of the study, surgical and anaesthesia care, including vital signs, complete and targeted physical assessments, laboratory tests, and ECGs will be performed at in accordance with normal hospital protocols. **There will be no deviation from standard care at any time point.**

All medical and surgical changes from baseline will be monitored throughout the study by the treating units and appropriate interventions will be taken accordingly.

The documentation of all adverse events specifically related to fluid intervention will begin at the time of first Trial Fluid administration and continue through to hospital discharge of all subjects involved in the study.

Data will be recorded in the CRF and will include a description of the event, date of onset, onset status (onset before/after the first dose of study medication), end of the event, severity, course of events, SAE, seriousness criteria, action with respect to study medication, treatment required, related to Trial Fluid and outcome of the event. AEs will be recorded and tabulated using the Common Terminology Criteria for Adverse Events (NCI-CTCAE) Version 4.0.

Severity of events will be determined by the Principal Investigators.

1.25 Definition of Adverse Events

An adverse event is defined as any untoward medical occurrence in a subject administered a study drug or has undergone study procedures and which does not necessarily have a causal relationship with this treatment. AE can therefore be any unfavourable and unintended sign (including an abnormal laboratory finding), symptom or disease temporally associated with the use of a medicinal (investigational) product, whether or not related to the medicinal (investigational) product.

Reporting of adverse events

The study Investigators will notify Austin Hospital Research Ethics Unit and Baxter Healthcare on an expedited basis and no later than the time set forth in the named regulations for each report of any adverse reaction that is "serious" and "unexpected" (as defined below) and believed by Austin Hospital and/or

the study Investigators to be causally associated with the use of the Study Materials.

A "serious adverse-reaction" is defined as any untoward medical occurrence that at any dose:

(a) results in death;
(b) is life threatening (i.e., the Study Subject was at immediate risk of death from the event as it occurred);
(c) is a persistent or significant disability/incapacity;
(d) requires inpatient hospitalization possibly related to the use of Study Materials;
(e) prolongs hospitalisation;
(f) is a congenital anomaly/birth defect; or
(g) is an important medical event that jeopardizes the Study Subject and requires medical/surgical intervention to prevent one of the outcomes listed in this definition.

An "unexpected adverse reaction" is defined as an event that is not identified in nature, severity, or frequency in the current investigator brochure/package insert/product information.

The study investigators will notify each other of any events or concerns about the safety of the Study Subjects that arise as the Study progresses, even if these events or concerns do not meet the definition of an adverse event/adverse reaction requiring notification as described above.

The Study Investigators shall send an expedited copy of each suspected unexpected serious adverse reaction (SUSAR) simultaneously to Baxter Healthcare at the time the Study Investigators sends it to Austin Hospitals Research Ethics Unit. In addition, the Study Investigators shall make available to Baxter Healthcare promptly such records as may be necessary and pertinent to investigate any such SUSAR, if specifically requested by Baxter Healthcare.

If a Baxter Healthcare product is used as part of the Study Materials, the Study Investigators shall notify Baxter Healthcare of all serious adverse events/adverse reactions (SAE) (including any pregnancy reports with or without an associated SAE) or other significant safety concerns (regardless of expectedness or causality) involving the Study Materials within 24 hours of awareness on a "CIOMS I" so that Baxter Healthcare may comply with worldwide regulatory authority reporting requirements related to the Baxter Healthcare product. The Study Investigators shall assist Baxter Healthcare in investigating any SAE and will provide any follow-up information requested by Baxter Healthcare.

Pregnancies shall be followed up by the Study Investigators to determine outcome (including premature termination) and status of mother and child and reported to Baxter Healthcare.

1.26 Reporting of Severe Adverse Events

In case of a SAE, the investigator will report the event to the Research Ethics Unit immediately (within 24 hours of awareness). The Chief investigator or delegate will complete and submit an SAE report form containing all information that is required on the form.

Reporting of a SAE to Baxter Healthcare does not relieve the Study Investigators of the responsibility for reporting it to the Austin Hospital Research Ethics Unit.

Any notification and reports required to be given to The Austin Hospital Research Ethics Unit, and to Baxter Healthcare and will be sent to:

Pharmacovigilance Manager
Baxter Healthcare Pty Limited
Telephone: +61 2 9848 1574
Facsimile: +61 2 9848 1123
Email: ANZProductSafety@baxter.com
Address: PO Box 88, Toongabbie, NSW, Australia, 2146

The Study Investigators shall ensure that the serious adverse event reporting and notification principles as agreed above are clearly reflected and described in this Protocol. In the event of any inconsistencies between the Protocol and the terms of this Agreement related to adverse event reporting, the terms of this Agreement shall prevail.

Whilst this study is an interventional study on the basis of conventional Guidelines, it has many features that are more akin to an observational study than an interventional one. Specifically, the study involves no departure from standard care and does not involve the collection of any data that are not already being collected for clinical and / or quality assurance purposes.

All of the events of interest noted above will be recorded on the CRF. Any situation involving these events of interest that also meets the criteria for an SAE will be recorded on the AE page of the CRF and marked ‘serious’.

Criteria for Causal Relationship of Trial Fluid

Causal relationship to Trial Fluid	Criteria for causal relationship
**Not Related**	A clinical event, including laboratory test abnormality, with a temporal relationship to the Trial fluid which makes a causal relationship improbable, and/or in which other drugs, chemicals or underlying disease or surgery provides plausible explanations.
**Possible**	A clinical event, including laboratory test abnormality, with a reasonable time sequence to administration of the Trial fluid, but which could also be explained by concurrent disease, or other drugs or chemicals or surgery.
**Probable**	A clinical event, including laboratory test abnormality, with a reasonable time sequence to administration of the Trial fluid, unlikely to be attributed to concurrent disease or other drugs or chemicals, or surgery.

1.27 Criteria for Causal Relationship to the Trial Fluid

Adverse events that fall under either "Possible" or "Probable" will be defined as "adverse events whose relationship to the Trial Fluid could not be ruled out".

1.28 Criteria for Defining the Severity of an Adverse Event

Severity of AEs will be graded according to Common Terminology Criteria for Adverse Events Version. The following guidelines will be used to determine grade:

Table 10: Criteria for Defining the Severity of a Trial Fluid Adverse Event Grade Description

Grade	Symptoms
1	**Mild;** asymptomatic or mild symptoms, clinical or diagnostic observations only; and intervention not indicated.
2	**Moderate;** minimal, local or non-invasive intervention indicated; limiting age; and appropriate instrumental activities of daily living.
3	**Severe or medically significant but not immediately life-threatening**; hospitalization or prolongation or hospitalization indicated; disabling; and limiting self-care activities of daily living.
4	**Life-threatening consequences**; and urgent intervention indicated.
5	**Death related to AE.**

STATISTICAL CONSIDERATIONS

1.29 Power calculations and sample size

A recently published open label sequential period study of chloride-liberal vs. chloride-restrictive fluid administration[2] demonstrated a reduction in the incidence of injury and failure based on RIFLE criteria from 14% (95% CI, 11% - 16%) to 8.4% (95% CI, 6.4% - 10%); P<0.001) with the introduction of a chloride-restrictive fluid regime.

Assuming that, as we have conservatively estimated, we can study 2000 subjects over a 24 week period, our sample size will provide more than 80% power with an alpha of 0.01 to detect a difference of this magnitude.

1.30 Analysis plan

A complete description of the statistical analyses will be specified in a statistical analyses plan, finalised prior to completion of the study.

All analyses will be conducted on an intention-to-treat basis.

The primary analyses will be unadjusted analyses in which binary outcomes will be compared using relative risks with 95% confidence intervals and chi square tests and continuous outcomes will be compared with the use of mean

differences and un-paired T-tests assuming that normality assumptions are meet. If normality assumptions are not met then we plan to attempt simple data transformation, such as a logarithm transformation, and if this fails to proceed to a Mann-Whitney rank based test. Adjusted analyses will be performed using Poisson regression for binary outcomes and linear regression for continuous outcomes. Baseline covariates will include age, gender, elective vs. emergency surgery, surgical specialty of admission, type of operation, and baseline serum creatinine level. Survival times will be compared using log-rank tests and presented as Kaplan-Meier curves.

1.31 Sub-groups

There will be the following subgroups:

- Patients undergoing abdominal surgery
- Patients undergoing vascular surgery
- Patients undergoing thoracic surgery
- Patients undergoing other surgeries (orthopaedics, urology etc.)
- Patients undergoing emergency surgery

1.32 Randomization process

Participants will be assigned to one of two groups, (Saline or Plasmalyte) using a random number allocation system (http://www.randomization.com).

1.33 2 Blinding

This is a double-blinded clinical trial. Blinding of all Trial fluids will be done by Baxter Healthcare Australia (BHA). BHA will compound the products in a strictly aseptic process. The shelf life of the solutions prepared will be set at 360 days, when stored below 25°C. Fluids will be prepared in 1 L clear plastic fluid container flasks (the exact same packaging that the fluids are normally prepared in). In order to differentiate between the three trial solutions, BHA will assign the Fluids with a unique code and batch number (Figure 1). Baxter will reveal the batch numbers corresponding to Fluids at the end of the data collection period. Each group will be assigned to a batch number, which matches the specific solution that the patient will be randomized to.

Importantly, study participants, and all involved in their medical and surgical treatment: surgeons, anaesthetists, anaesthesia nurses and all clinical staff involved in the care of each participant will blinded to treatment allocation.

Figure 1. Labelling on blinded trial fluid bag showing label information including batch numbers

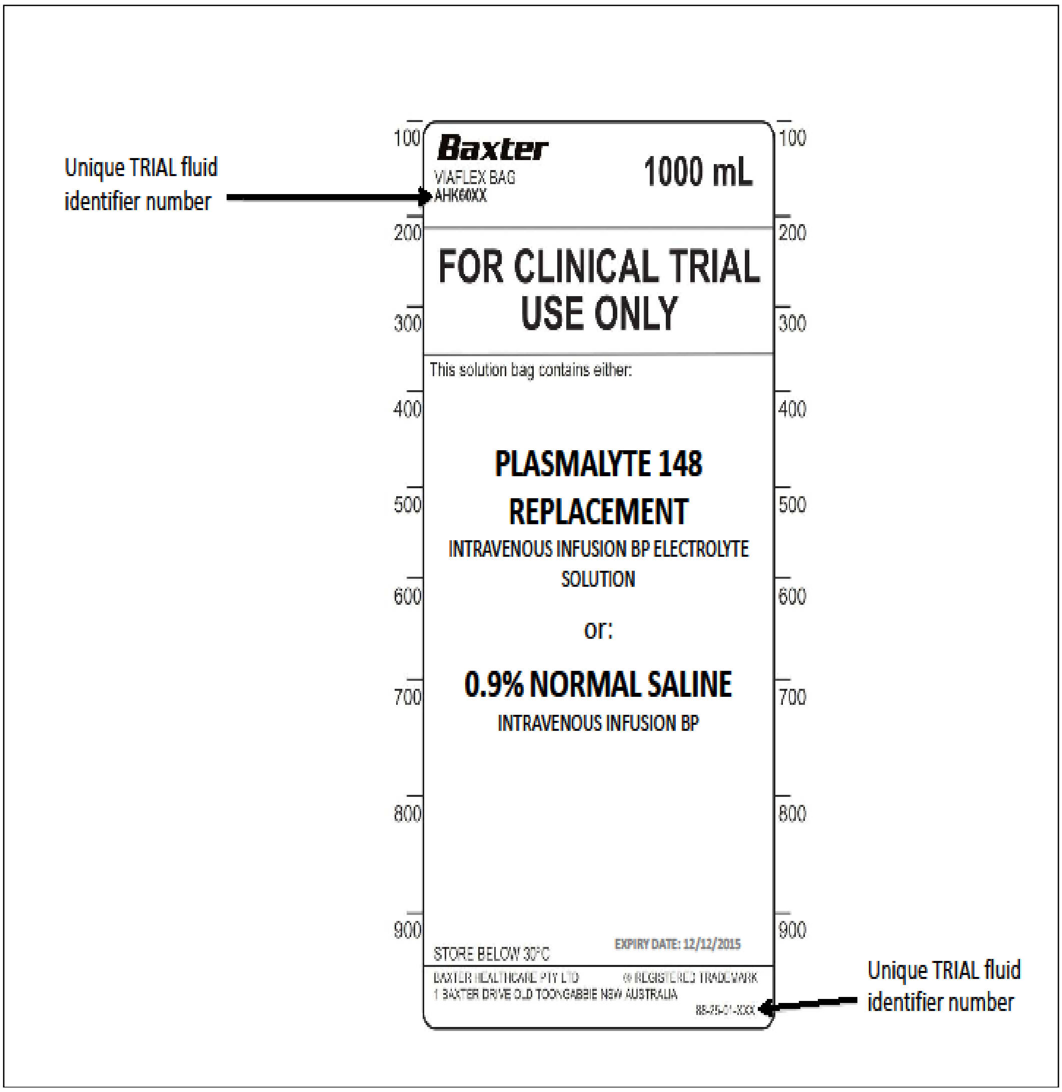

STUDY BUDGET

1.34 Detailed budget

Item	Year 1
**Compounding of trial fluid solutions:**<br>**Cost: $1.16 per litre for Saline (0.9%)**<br>**Cost: $1.77 per litre Plasmalyte**<br>Assuming 1000 patients each group each requiring 10L fluid each<br>Total Fluids:<br>Plasmalyte: 10 000 Litres<br>Saline (0.9%): 10 000 Litres	 <br><br><br><br>$17 700<br>$11 600
**Computer consumables (printing, paper, ink)**<br>**Professional statistical consultation**<br>**Application to TGA for CTN**	$1000<br>$3000<br>$300
**Total (AUD)**	**$ 33,600**

1.35 Dispensing and storage costs

There will be no additional costs incurred for the dispensing of the Trial fluids by Austin Hospital pharmacy beyond that which is already part of normal dispensing practices. Mr Kent Garrett will be responsible for the supply of fluids to the allocated areas in theatres, intensive care and the surgical wards.

Baxter Healthcare will deliver all fluids to Austin Hospital as part of normal Hospital protocols and fluid will be stored in Austin Stores in accordance with normal pharmacy practices.

Baxter Healthcare will be responsible for the cost of compounding of all the trial fluids.

STUDY ADMINISTRATION STRUCTURE

1.36 Department of Anaesthesia & Intensive Care responsibilities

- Overall management of the study will be by both the Department of Anaesthesia and Intensive Care at Austin Hospital
- Both departments will be involved in the case report form design and production
- Both departments will provide assistance for training for Research Co-ordinators and study team if necessary
- Co-ordination of delivery of study fluids with be under the care of Mr Kent Garrrett, Director of Pharmacy at Austin Hospital
- Study database set-up and co-ordination of data entry will be conducted by all principle investigators

1.37 Data management responsibilities

- Data queries
- Data analysis

1.38 Management committee responsibilities

- The Principle Investigators will liaise with all surgical units in the introduction of this trial. In additional, all ward nursing staff, resident medical officers and clinicians caring for these patients will be informed and educated about the study rationale and design.

FUNDING

This study will be submitted for funding to the Australian and New Zealand College of Anaesthesia.

PUBLICATIONS

The study will be published in the name of the of the study investigators. The chief investigator will be listed as the first author and other members of the management committee will be listed alphabetically. Funding bodies will be acknowledged in the publication

REFERENCES

1. Finfer S, Liu B, Taylor C, et al. Resuscitation fluid use in critically ill adults: an international cross-sectional study in 391 intensive care units. Crit Care 2010;14:R185.
2. Yunos NM, Bellomo R, Hegarty C, Story D, Ho L, Bailey M. Association between a chloride-liberal vs chloride-restrictive intravenous fluid administration strategy and kidney injury in critically ill adults. JAMA : the journal of the American Medical Association 2012;308:1566-72.
3. Yunos NM, Bellomo R, Story D, Kellum J. Bench-to-bedside review: Chloride in critical illness. Crit Care 2010;14:226.
4. Waikar SS, Winkelmayer WC. Saving the kidneys by sparing intravenous chloride? JAMA : the journal of the American Medical Association 2012;308:1583-5.
5. Ropper AH. Hyperosmolar therapy for raised intracranial pressure. The New England journal of medicine 2012;367:746-52.
6. Bellomo R, Ronco C, Kellum JA, Mehta RL, Palevsky P. Acute renal failure - definition, outcome measures, animal models, fluid therapy and information technology needs: the Second International Consensus Conference of the Acute Dialysis Quality Initiative (ADQI) Group. Crit Care 2004;8:R204-12.
7. Stow PJ, Hart GK, Higlett T, et al. Development and implementation of a high-quality clinical database: the Australian and New Zealand Intensive Care Society Adult Patient Database. Journal of critical care 2006;21:133-41.
8. Kellum JA, Song M, Li J. Science review: extracellular acidosis and the immune response: clinical and physiologic implications. Crit Care 2004;8:331-6.
9. Williams EL, Hildebrand KL, McCormick SA, Bedel MJ. The effect of intravenous lactated Ringer's solution versus sodium chloride solution on serum osmolality in human volunteers. Anesthesia and analgesia 1999;88:999-1003.
10. Chowdhury AH, Cox EF, Francis ST, Lobo DN. A randomized, controlled, double-blind crossover study on the effects of 2-L infusions of saline and plasma-lyte(R) 148 on renal blood flow velocity and renal cortical tissue perfusion in healthy volunteers. Annals of surgery 2012;256:18-24.
11. Wakim KG. "Normal" 0.9 per cent salt solution is neither "normal" nor physiological. JAMA : the journal of the American Medical Association 1970;214:1710.
12. Guidet B, Soni N, Della Rocca G, et al. A balanced view of balanced solutions. Crit Care 2010;14:325.
13. Liu B, Finfer S. Intravenous fluids in adults undergoing surgery. BMJ 2009;338:b2418.

14. Shaw AD, Bagshaw SM, Goldstein SL, et al. Major complications, mortality, and resource utilization after open abdominal surgery: saline compared to Plasma-Lyte. Annals of surgery 2012;255:821-9.
15. Davies PG, Venkatesh B, Morgan TJ, Presneill JJ, Kruger PS, Thomas BJ, Roberts MS, Mundy J. Plasma acetate, gluconate and interleukin-6 profiles during and after cardiopulmonary bypass: a comparison of Plasma-Lyte 148 with a bicarbonate-balanced solution. Crit Care. 2011;15(1):R21. doi: 10.1186/cc9966. Epub 2011 Jan 14.
16. Bingel M, Lonnemann G, Koch KM, Dinarello CA, Shaldon S: Enhancement of in-vitro human interleukin-1 production by sodium acetate. Lancet 1987, 1:14-16.
17. Thaha M, Yogiantoro M, Soewanto, Pranawa: Correlation between intradialytic hypotension in patients undergoing routine hemodialysis and use of acetate compared in bicarbonate dialysate. Acta Med Indones 2005, 37:145-148.
18. Veech RL, Gitomer WL: The medical and metabolic consequences of administration of sodium acetate. Adv Enzyme Regul 1988, 27:313-343.
19. Schrander-vd Meer AM, ter Wee PM, Kan G, Donker AJ, van Dorp WT: Improved cardiovascular variables during acetate free biofiltration. Clin Nephrol 1999, 51:304-309.
20. Quebbeman EJ, Maierhofer WJ, Piering WF: Mechanisms producing hypoxemia during hemodialysis. Crit Care Med 1984, 12:359-363.
21. Jacob AD, Elkins N, Reiss OK, Chan L, Shapiro JI: Effects of acetate on energy metabolism and function in the isolated perfused rat heart. Kidney Int 1997, 52:755-760.
22. Selby NM, Fluck RJ, Taal MW, McIntyre CW: Effects of acetate-free double-chamber hemodiafiltration and standard dialysis on systemic hemodynamics and troponin T levels. ASAIO J 2006, 52:62-69.
23. Traverso LW, Hollenbach SJ, Bolin RB, Langford MJ, DeGuzman LR: Fluid resuscitation after an otherwise fatal hemorrhage: II. Colloid solutions. J Trauma 1986, 26:176-182.\
24. Westaby S: Organ dysfunction after cardiopulmonary bypass. A systemic inflammatory reaction initiated by the extracorporeal circuit. Intensive Care Med 1987, 13:89-95.
25. Scheingraber S, Rehm M, Sehmisch C, Finsterer U: Rapid saline infusion produces hyperchloremic acidosis in patients undergoing gynecologic surgery. Anesthesiology 1999; 90: 1265-70
26. Hadimioglu N, Saadawy I, Saglam T, Zeki Ertug Z, Dinckan A: The Effect of Different Crystalloid Solutions on Acid-Base Balance and Early Kidney Function After Kidney Transplantation. Anesthesia and Analgesia 2008; 107: 264-269
27. Liskaser F, Story DA, Hayhoe M, Poustie SJ, Bailey MJ, Bellomo R: Effect of pump prime on acidosis, strong-ion-difference and unmeasured ions during cardiopulmonary bypass. Anaesth Intensive Care 2009; 37: 767-72
28. Liskaser FJ, Bellomo R, Hayhoe M, Story D, Poustie S, Smith B, Letis A, Bennett M: Role of pump prime in the etiology and pathogenesis of

cardiopulmonary bypass-associated acidosis. Anesthesiology 2000; 93: 1170-3
29. Powell-Tuck J, Gosling P, Lobo DN, Allison SP, Carlson GL, Gore M, Lewington AJ, Pearse RM, Mythen MG: British Consensus Guidelines on Intravenous Fluid Therapy for Adult Surgical Patients (GIFTASUP), 2009
30. Watanabe I, Mayumi T, Arashima T, Takahashi H, Shikano T, Nakao A, Nagino M, Nimura Y, Takezawa J: Hyperlactaemia can predict the prognosis after liver resection. Shock 2007; 28: 35-38
31. Jansen TC, van Bommel J, Bakker J: Blood lactate monitoring in critically ill patients: a systematic health technology assessment. Crit Care Med 2009; 37: 2827-39
32. Chhibber A, Dziak J, Kolano J, Norton JR, Lustik S: Anesthesia care for adult live donor hepatectomy: our experiences with 100 cases. Liver Transpl 2007; 13: 537-542
33. Myburgh JA, Finfer S, Bellomo R, et al. Hydroxyethyl Starch or Saline for Fluid Resuscitation in Intensive Care. The New England journal of medicine 2012.
34. Weinberg L, Sullivan R, Siu L, Scurrah N, Nikfarjam M, McNicol L, Bellomo R, Story D. A multicentre randomized double-blind controlled non-inferiority multicentre study of Plasmalyte vs. Compound Lactate Solution (Hartmann's solution) in patients receiving liver resection (Australian New Zealand Clinical Trials Registry No: 12610000147088)
35. Story DA, Lees L, Weinberg L, Teoh SY, Lee KJ, Velissaris S, Bellomo R, Wilson SJ. Anesthesiology. 2013 Sep;119(3):569-575.Cognitive Changes after Saline or Plasmalyte Infusion in Healthy Volunteers: A Multiple Blinded, Randomized, Cross-over Trial.
36. Weinberg L, Liskaser F, Story D, Bellomo R. Effect of pump prime on acidosis, strong-ion-difference and unmeasured ions during cardiopulmonary bypass: a randomized blinded clinical trial. (Australian New Zealand Clinical Trials Registry No: 12612000022864)
37. Weinberg L, Story D, Bellomo R. Effect of Plasmalyte or Normal saline on acidosis, strong-ion-difference and unmeasured ions in patients undergoing cadaveric renal transplantation (Study in Progress).
38. Finfer S, Chittock DR, Su SY, et al. Intensive versus conventional glucose control in critically ill patients. The New England journal of medicine 2009;360:1283-97.
39. Finfer S, Liu B, Chittock DR, et al. Hypoglycemia and risk of death in critically ill patients. The New England journal of medicine 2012;367:1108-18.
40. Cooper DJ, Rosenfeld JV, Murray L, et al. Decompressive craniectomy in diffuse traumatic brain injury. The New England journal of medicine 2011;364:1493-502.
41. Ranieri VM, Thompson BT, Barie PS, et al. Drotrecogin alfa (activated) in adults with septic shock. The New England journal of medicine 2012;366:2055-64.

42. Bellomo R, Cass A, Cole L, et al. Intensity of continuous renal-replacement therapy in critically ill patients. The New England journal of medicine 2009;361:1627-38.
43. Finfer S, Bellomo R, Boyce N, French J, Myburgh J, Norton R; SAFE Study Investigators. A comparison of albumin and saline for fluid resuscitation in the intensive care unit. N Engl J Med. 2004 May 27;350(22):2247-56.
44. Knaus WA, Wagner DP, Draper EA, et al. The APACHE III prognostic system. Risk prediction of hospital mortality for critically ill hospitalized adults. Chest 1991;100:1619-36.
