# Supplementary material for: The effects of 0.9% saline versus Plasma-Lyte 148 on renal function as assessed by creatinine concentration in patients undergoing major surgery: A single-centre double-blinded cluster crossover trial
Source: PLoS One. 2021 May 19;16(5):e0251718. doi: 10.1371/journal.pone.0251718 (PMC8133498; doi:10.1371/journal.pone.0251718)
Supplement: Attachment — Submitted filename: RESPONSE TO REVIEWERS_PLOS_ONE_22_Feb_2021.pdf [file pone.0251718.s008.pdf]

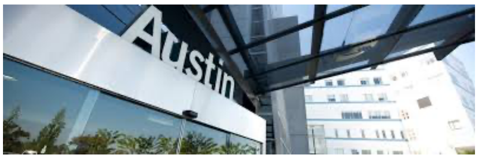

Austin Health

A/Prof Laurence Weinberg

Director
DEPARTMENT OF ANAESTHESIA
AUSTIN HEALTH

Tel: (03) 9496 3800 Fax: (03) 9459 6421
Email: Laurence.Weinberg@austin.org.au

Director of Anesthesia:
A/Prof Laurence Weinberg
BSc, MBBCh, MRCP,
FANZCA, MD

Admin Managers:
Mrs Val Hocking
Mrs Helen Pitman

Staff anaesthetists:
Dr Dishan Chandrasekara
MBBS, FANZCA
Dr Chong Tan
MBBS, FANZCA
Dr Louise Ellard
MBBS, FANZCA
Dr Brett Pearce
MBBS, FANZCA
Dr Raymond Hu
MBBS, FANZCA
Dr Audrey Bren
MBBS, FANZCA
Dr Lachlan Miles
MBBS, FANZCA
Dr Dean Cowie
MBBS, FANZCA
Dr Jonathan Fernandes
MBBS, FANZCA
Dr Jonathan Graham
MBBS, FANZCA
Dr Ian Harley
MBBS, FANZCA, FRCA
Dr William Howard
MBBS, Dip Med(Pain Mgt),
FANZCA, FFPMANZCA
Dr Antony Leaver
MBBS, FANZCA
Dr Frank Liskaser
MBBS, FANZCA
Dr Shiva Malekzadeh
MBBS, FANZCA
Dr Peter McCall
MBBS, FANZCA;
A/Prof Philip Peyton
MBBS, FANZCA, DA(UK),
DipObs(RACOG), MD
Dr Param Pillai
MBBS, MD Anes, FRCA
Dr Nick Scurrah
MBBS, FANZCA
Prof David Story
MBBS, FANZCA, MD
Dr David Tremewen
MBBS, FANZCA

RESPONSE TO REVIEWERS

PONE-D-20-37578
The effects of 0.9% saline versus Plasma-Lyte 148 on renal function as assessed by creatinine concentrations in patients undergoing major surgery: a single-centre double-blinded cluster crossover trial

On behalf of my co-authors, I would like to sincerely thank each Reviewer for considering our revised manuscript for publication. The comprehensive reviews and constructive comments provided by yourself and the expert reviewers have been immensely appreciated. The insightful and constructive comments have enabled us to strengthen the scientific rigor and merits of our research. Thank you once again for considering our resubmission for publication.

Reviewer #1

We thank Professor Fernando Zampieri for his valuable and constructive comments. Further we appreciate the time taken to review our manuscript

Reviewer #1, Question 1. This is one of the very fortunate cases where the reviewer job is easy. This is a solid pilot trial, well conducted, clearly reported and that is of crucial importance for larger endeavours. Data reporting is clear. The observed imbalances are accounted for in the methods and discussion. The primary endpoint is properly analysed.
Question 1. Authors' response: Thank you for your positive comments. Once again, we thank you taking the time to review our manuscript.

Reviewer #1, Question 2. My only (very very minor) comment is that I would not be that sure if chloride is a possible culprit for AKI, so perhaps just lower the tone a bit by saying that balanced fluids may be associated with less AKI and that chloride could be related to it. I would specially avoid making statements about chloride and AKI in the abstract.
Question 2. Authors' response: Thank you for these important comments. In the abstract, we have removed all references about any association with chloride and AKI. In the "Objectives" section we now state "*In a double-blind cluster crossover study comparing the renal effects of saline and Plasma-Lyte for fluid therapy in patients undergoing major elective and emergency surgery, we found that the study design was feasible to support a future follow-up larger clinical trial. Both patient and clinician acceptance were excellent. We observed no statistically significant difference in the incidence of postoperative AKI (as measured by changes in serum creatinine) or other postoperative complications.*"

Reviewer #2

We thank **Reviewer #2** for their valuable time taken to review our manuscript. Please find below a detailed response to each of the important comments.

Reviewer #2: Important note: This review pertains only to 'statistical aspects' of the study and so 'clinical aspects' [like medical importance, relevance of the study, 'clinical significance and implication(s)' of the whole study, etc.] are to be evaluated [should be assessed] separately/independently.

Reviewer #2: Question 1. COMMENTS: Actually, your ABSTRACT is well drafted but assay type [though you started with 'Background', I guess with intention to divide the ABSTRACT in sections]. Please note that it is preferable [refer to item 1b of CONSORT checklist 2010: Structured summary of trial design, methods, results, and conclusions] to divide the ABSTRACT with small sections like 'Objective(s)', 'Methods', 'Results', 'Conclusions', etc. which is an accepted practice of most of the good/standard journals [including this one]. It will definitely be more informative then, I think, whatever the article type may be.
Authors' response, Question 1. Thank you for this important comment. We have now followed the CONSORT checklist and restructured the Abstract into the following 4 headings: Objectives, Methods, Results, and Conclusions.

Reviewer #2: Question 2. I very strongly feel (and so have a very serious objection) that inclusion of the term 'crossover trial' is by [I guess] oversight/mistake. Note that the cross-over design {as per my knowledge of 'standard definition' and 'ideal/original concept'} is a special case of a randomized control trial that allows each subject to serve as his own control. Following is a quote from a standard text-book: *In order to use the cross–over design, assumption to be made is that the effect of the intervention during the first period must not carry–over into the second period (carry–over effect or spill-over effect). Therefore, it is necessary that the therapies under study have no carry–over effect. Differential carry-over effect may be eliminated by interposing a long dry–out or wash–out period (the length of which is determined by the pharmacological properties of the drugs being tested) between the termination of the treatment given first and the beginning of the treatment given second.*

I am sure that the authors know above quoted things. However, please specify clearly if you intended to imply something else (some other concept) by the term 'crossover trial' [remember that this is a scientific/academic document and so all details should be clearly/correctly communicated]. In fact, use of (standard) crossover trial in the field of 'surgery' is rare and so is highly appreciated [if it is correctly adapted/used]. Further read the following paragraph from the same book: *The appeal of this design is to avoid 'between – subject' variation in estimating the intervention effect, thereby all patients can be assured that sometime during the course of investigation, they will receive the new therapy {both treatments}. However, this method of study is not suitable if the drug of interest cures the disease, if the drug is effective only during a certain stage of the disease or if the disease changes radically during the period of time required for the study.*

Authors' response, Question 2. That you for this excellent comment. We strongly agree with your comments about a "cross-over design" study. This is a very important point, however it's important to appreciate that the design of our study is not a conventional "cross-over trial", rather, as we have clearly stated in the title, abstract and the methods sections of the manuscript, our design is a "cluster crossover trial".

There are fundamental and important differences in the analysis of a “cross-over design study” and a “cluster crossover trial” (1). In our study the whole “cluster” of individuals (i.e. the hospital), rather than the individual patients, has been crossed over from treatment A to treatment B with the order of treatments being randomly chosen. All patients admitted to our single institution over the predefined 6-week period can therefore be thought of as belonging to a cluster. The cluster cross-over nature of our study would have perhaps been clearer if multiple clusters/centres were to be crossed-over such as in, e.g., a stepped-wedge design, but the fact that in our study only a single cluster underwent a cross-over does not discount the single cluster single cross-over nature of the study.

The statistical analysis applied for our cluster crossover trial are therefore appropriate.

Reference
[1]Arnup SJ, McKenzie JE, Hemming K, Pilcher D, Forbes AB. Understanding the cluster randomised crossover design: a graphical illustration of the components of variation and a sample size tutorial. Trials. 2017 Aug 15;18(1):381. Doi: 10.1186/s13063-017-2113-2. PMID: 28810895; PMCID: PMC5557529.

Reviewer #2: Question 3. In table-2, you compare two treatment [Plasma-Lyte vs Saline after surgery] with different sample (group) sizes [in real crossover trial the group sizes are same as each subject will receive both treatments ultimately (only in different ‘order’)]. One known variation is ‘case-crossover’ design. This design is useful when the risk factor/exposure is transient. Data yielded by this design are analyzed as of ‘matched case-control’ studies. I do not see any of these [concept used as per standard] anywhere in this manuscript.
Authors’ response, Question 3. As we have stated above, given that we have performed a “cluster crossover trial”, we believe our statistical analyses are appropriate.

Reviewer #2, Question 4. All the methodologically important concepts [like double-blinding, clustering, crossover trial] adapted/used here are not made clear. Account given {example regarding double-blinding described in lines 104-118 & also figure-1 regarding ‘Cluster crossover design of Saline vs Plasma-Lyte study’ does not depict clearly the ‘crossover’ nature, it mainly gives ‘time line’} is ‘confusing’, I feel. Advantages generally known of double-blinding are applicable for “parallel independent groups” design. For ‘crossover’ design (except avoidance of ‘auto- and hetero-suggestion’ bias) they may differ, I guess.
Authors’ response, Question 4. Thank you for this excellent comment. We have now included an additional statement in the methods section clarifying what a “cluster crossover trial” is. We agree that this will be important for the reader who is not familiar with this type of specific trial design. Further, we have made minor modifications to Figure 1, to allow the reader to better appreciate the cluster crossover design. In the Methods section, we also have included the following an additional statement “*Unlike a conventional randomized clinical trial, “clusters” of individuals, rather than the individual patients were blindly allocated to receive either saline or Plasma-Lyte”.*

We have also made some minor modification to Figure 1 to provide the reader with more clarity regarding the cluster crossover trial design.

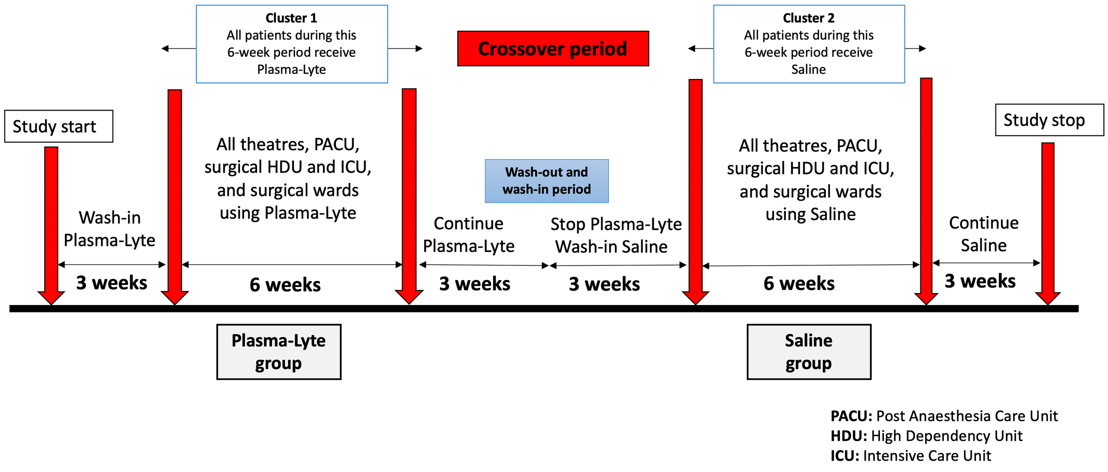

Reviewer #2, Question 5. Moreover, all 'statistical analyses' performed here [example, line 164-65: Comparisons between categorical variables were made using chi-square and Fisher's exact tests, while continuous variables were compared using the Mann–Whitney U test. Including estimation of IRR, HR, all 95%Cis] assume 'independence of the groups/samples' which is violated in case 'crossover trial'.
Authors' response, Question 5. As we have stated above, given that we have performed a "cluster crossover trial" with a single cluster and not an individual patient "cross-over trial", we believe our statistical analysis are appropriate.

Reviewer #2, Question 6. In my opinion, it [the ethics committee granting waiver of participant consent] is not a standard (practice) for any reason [like given in lines 77-79: participation in the research posed no more than minimal increase in risk to individuals than what they would be exposed to if they were not in the study, the ethics committee granted waiver of participant consent]. Other analyses {as described in lines 172-180 (We conducted time-to-event analyses using Cox proportional hazard regression models, and compared non-dichotomous outcomes using Poisson or negative binomial regression models, all adjusted for the same set of covariates, with treatment effects reported as adjusted hazard ratios with 95% Cis. The threshold for statistical significance was a P value of 0.05. All secondary and exploratory endpoints are reported as point estimates of treatment effects with 95% Cis.29 We report both covariate-adjusted and unadjusted outcomes. We used forest plots to present the primary and key secondary outcomes regarding the consistency of a treatment effect across the subgroups)} are alright.
Authors' response, Question 6. Thank you for the positive comments regarding our use of these statistical tests.

Reviewer #2, Question 7. Fig 2. CONSORT diagram and Fig 3. Incidence rate ratios of key outcomes for patients receiving Plasma-Lyte compared to Saline are also good.
Authors' response, Question 7. Thank you for these positive comments.

Reviewer #2, Question 8. Overall, this article needs to be re-drafted and also change in title considering above highlighted points, I guess.
Authors' response, Question 8. We hope our responses have assured the Reviewer that our study is not a "cross-over trial", rather a "cluster crossover trial". Therefore, we believe our statistical analyses are appropriate.

Reviewer #3

We thank **Reviewer #3** for their valuable comments and time taken to review our manuscript. Further we appreciate this expert and considered review. Please find below a detailed response to each of these important comments.

Reviewer #3: Question 1. General Comments: In a single-cluster, single-crossover clinical trial, the authors compare administration of Plasma-Lyte versus 0.9% sodium chloride (saline) among adults undergoing surgery lasting more than two hours and requiring post-operative hospitalization for at least one night with regard to the primary outcome of AKI by RIFLE creatinine criteria. They report similar incidence of AKI (8.1% vs 10.7%) and complications but slightly shorter hospital length of stay with Plasma-Lyte compared to saline.

Strengths include:
(1) importance of the research question;
(2) control of study group assignment by the trial;
(3) control of fluid in the operating room, ICUs, and hospital wards.

Weaknesses include:
(1) the inability of a single-cluster single-crossover design to address cluster and period effects;
(2) a population with a relatively low risk of any stage of AKI;
(3) a relatively small average volume of fluid; and
(3) presentation of the manuscript in a manner that de-emphasizes the pilot endpoints while over-emphasizing the clinical endpoints and incomplete description of the study population.

Authors' response, Question 1. Thank you for highlighting the key strengths and weaknesses of the manuscript. We hope that our detailed responses and modification to these insightful questions enhance the scientific merits of this study and address and strengthen some of the weaknesses highlighted above.

Reviewer #3: Major Comments:

Reviewer #3: Question 1. Study population – The description of the methods in the text suggest that the blinded fluid assigned to a given period was applied to all patients in the OR, ICUs, and wards during the study period...and then only those who met the study's inclusion criteria were analyzed (i.e., patients could have received blinded study fluid and not be in the analysis). Is this correct? If so, this is reasonable for a comparison of the effectiveness of two commonly used interventions, but the methods and CONSORT diagram should make this clear. Specifically, did the 4,586 patients excluded from the primary analytic population because they had a surgical duration < 2 hours receive the blinded study fluid assigned to that cluster? If so, it is inaccurate to describe them as excluded from the trial prior to study group assignment. These patients should be excluded from each respective group AFTER allocation. This is important, in part, because this trial appears to have a primary analytic population that was defined using post-enrolment factors (e.g., duration of surgery – something that cannot be determined prior to enrolment). This is usually a major problem in randomized trials, as study group assignment might then impact which patients meet the eligibility criteria, re-introducing selection bias (which randomization usually mitigates).
Authors' response, Question 1. The reviewer has made some excellent points, all of which we agree with. Importantly, as the Reviewer has stated, for pragmatic and logistical

reasons, blinded fluid was assigned for a given period and administered to all patients undergoing surgery during that period. We then “included” or “excluded” patients based on prespecified trial criteria, which have been outlined in our manuscript. The fluid was then continued in all patients in the intensive care unit and all patients on the ward. Importantly, we only analysed patients who met the study’s inclusion criteria.

We also agree with Reviewer that this trial has a primary analytic population defined using post-enrolment factors (e.g., duration of surgery), which as the Reviewer has correctly stated, could not be determined prior to enrolment. We also agree that this can be a concern in randomized trials, as study group assignment can potentially impact on which patients meet the eligibility criteria, re-introducing selection bias (which randomization usually mitigates). However, for our study, we acknowledge that this is **“theoretically”** possible i.e., that the intervention could bias the inclusion and exclusion criteria, however in reality, there is no causality whatsoever between the treatment and the inclusion/exclusion criteria, all of which we prespecified. We assure the reviewer that the treatment i.e., saline or Plasmalyte does bias patient inclusion or patient exclusion.

In response to the Reviewer’s comments, in the Results section, we now state “*In total, 5646 patients were screened over the duration of the two predefined cluster periods. During the first 6-week cluster period, 2933 patients were allocated to receive saline; during the second 6-week cluster period, 2713 patients were allocated to receive Plasma-Lyte. After exclusions, 1060 patients fulfilled the inclusion criteria – 602 patients were allocated to the saline group and 458 to the Plasma-Lyte group”.*

In addition, our CONSORT diagram has now been corrected to accurately reflect this important point. Thank you once again for these excellent suggestions.

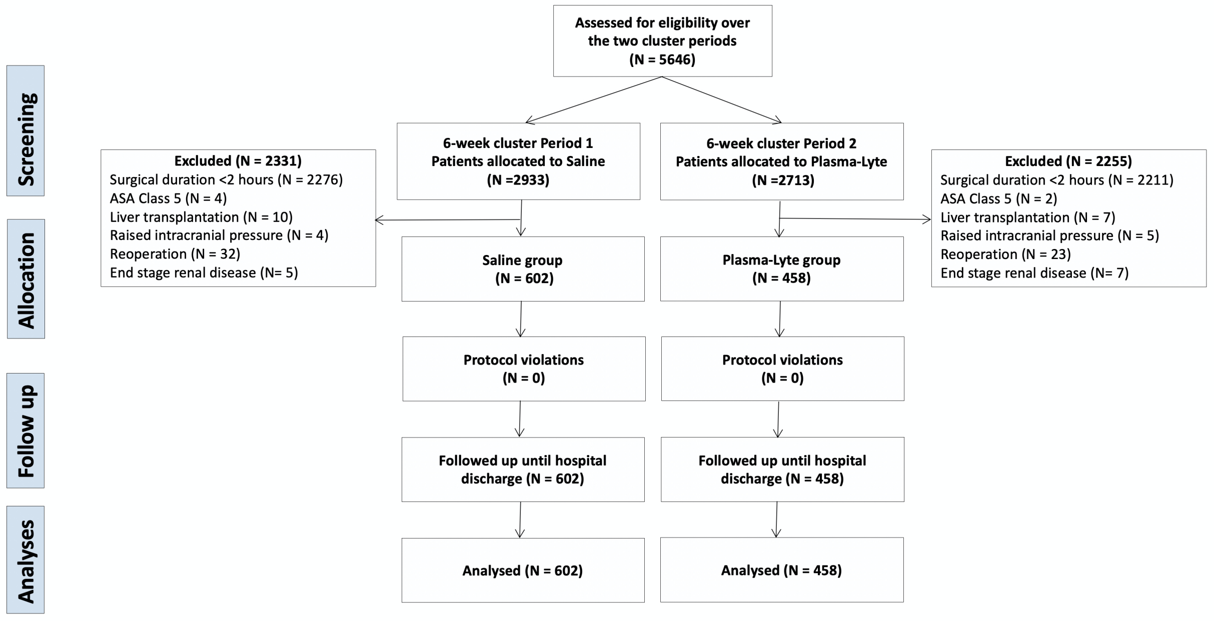

Reviewer #3: Question 2. To convince the readers that use of post-enrolment eligibility criteria did not bias the analysis, please consider: Moving all post-enrolment exclusions below allocation in the consort and confirming that the proportion of patients excluded for each criteria after group assignment did not differ between the two study groups
Authors' response, Question 2. We have now moved all post-enrolment exclusions below allocation in the Consort diagram. Kindly refer to the revised Consort diagram presented above. We did not analyse differences between the proportions of patients excluded from each cluster period. This was not the proposed intention of the study and we did not have sufficient funding nor the resources to undertake this. We do not think this compromises the integrity of our findings. We have now acknowledged this as a limitation of the study in the discussion section.

Reviewer #3: Question 3. Adding (if available) a sensitivity analysis of the primary outcome among ALL patients who received the blinded study fluid – not limited to those who met post-enrolment eligibility criteria. (If I have misunderstood the application of these eligibility criteria and they were truly assessed prior to enrolment and patients with shorter surgical durations or patients in the ICU who had not undergone a qualifying operation did NOT receive study fluid, please ignore this comment, but clarify in the methods).
Authors' response, Question 3. We did not analyse differences between the proportions of patients excluded from each cluster period. Neither did we undertake a sensitivity analysis of the primary outcome among all patients who received the blinded study fluid i.e., not limited to those who met post-enrolment eligibility criteria. We have acknowledged these limitations of the study and outlined these in the discussion section.

Reviewer #3: Question 4. Outcomes – Under Methods the Primary Aim of this study was described as "to establish the pilot feasibility, safety, and preliminary efficacy evidence base for a large trial". In the results section and conclusions, however, the authors focus on the clinical endpoints as if the primary aim of the trial were to guide clinical care (like the large trial they hope this pilot will support). For example, the first paragraph of the discussion's "key finding" is "our findings suggest that clinicians can reasonably use either solution intraoperatively...". In the conclusion they state "these findings can inform clinician's decision regarding which type of crystalloid fluid to use for patients receiving major surgery."

A pilot trial aimed at establishing feasibility and safety of a larger trial (with a single-cluster single-crossover design that does not balance baseline characteristics or address cluster and period effects) should NOT be used to inform clinicians decision making in the treatment of patients! This is an excellent pilot study, but the authors must revise the results and discussion/conclusion to emphasize the feasibility, safety, and preliminary efficacy aims that they set out to address.

Patient acceptance? What information informs the safety of a larger trial? What measures suggest this design is feasible? Enrolment rate? Compliance? Crossover or contamination? Rates of the outcome overall? Rare occurrence of the exclusion criteria?
Clinician acceptance? Patient acceptance? What information informs the safety of a larger trial?

Please present any information relevant to these assessments in the results.

Authors' response, Question 4. Thank you for these excellent points that we agree with. Accordingly, we have revised the results, discussion and conclusion section of the study and included detailed information on study design feasibility, enrolment rate, compliance, crossover or contamination, rates of the outcome overall, occurrence of the exclusion criteria, and clinician and patient acceptance.

We hope that we have addressed this point in detail. Below is a brief overview of the additional changes made.

1. Abstract. We have included the following statement "*The primary aim was to establish the pilot feasibility, safety and preliminary efficacy evidence base for a large interventional trial to establish whether saline or Plasma-Lyte is the preferred crystalloid fluid for managing major surgery patients. The primary efficacy outcome was the proportion of patients with changes in renal function as assessed by creatinine concentrations during their index hospital admission. We used changes in creatinine to define acute kidney injury (AKI) according to the RIFLE criteria."*

   The results of the abstract also now address feasibility end points, efficacy end pints and safety end points. We now state "*The study was feasible with 100% patient and clinician acceptance. There were no deviations from the trial protocol.*" The results conclude with the statement "*There were no serious adverse events that related to the specific type of fluid received and there were no fluid crossover or contamination events."*

   The conclusion has been modified reflect feasibility, efficacy and safety. We now state "*The study design was feasible to support a future follow-up larger clinical trial. Patients treated with saline did not demonstrate an increased incidence of postoperative AKI (defined as changes in creatinine) compared to those treated with Plasma-Lyte. Our findings imply that clinicians can reasonably use either solution intraoperatively for adult patients undergoing major surgery."*

2. In the methods section we now briefly describe and overview the logistical and operation challenges, with reference to fluid governance, storage and delivery to all clinical areas.

   Further in the methods section we state "*Feasibility outcomes included patient and clinician acceptance, recruitment rate, reasons for exclusion, logistical or operation feasibility with fluid storage and delivery to the clinical areas where the trials was undertaken, deviations from trial protocol, unblinding rate, and number of patients who fulfilled eligibility who did not have renal function measured as part of routine clinical care."*

   The primary efficacy outcome was the proportion of patients with AKI defined by creatinine levels and assessed according to the risk, injury, failure, loss of kidney function and end-stage renal failure (RIFLE) criteria during the index hospital admission.

   We also state that "Safety outcomes included fluid administration errors, unintentional fluid discontinuation, crossover or contamination events.

3. Results. We systematically present the results of feasibility, efficacy and safety as outlined above.

4. Conclusion section: As suggested we now address feasibility, preliminary efficacy and safety. We now conclude by stating *"In a double-blind cluster crossover study comparing the renal effects of saline and Plasma-Lyte for fluid therapy in patients undergoing major elective and emergency surgery, we found that study design was feasible to support a future follow-up larger clinical trial. Both patient and clinician acceptance were excellent. We observed no statistically significant*

difference in the incidence of postoperative AKI (as measured by changes in serum creatinine and eGFR), or other postoperative complications.

Thank you once again for these excellent comments and insights. They are very much appreciated.

Reviewer #3: Question 5. Please revise the discussion and conclusion to give the author's assessment, as the one's who know this trial best, on how this trial does inform the design of future trials on this topic in the field. How should a larger trial be designed similarly or differently? Population? Outcomes? Duration of intervention? Type of surgery?
Authors' response, Question 5. Thank you for this constructive comment. We have now made significant modifications to the discussion of the manuscript reflecting the above points.

Reviewer #3: Question 6. Please also make sure these changes to focus on the feasibility and safety aims of this pilot trial are reflected in the abstract.
Authors' response, Question 6. Thank you for this important comment.

We have made the following changes to the Abstract. We include the following statement "*The primary aim was to establish the pilot feasibility, safety and preliminary efficacy evidence base for a large interventional trial to establish whether saline or Plasma-Lyte is the preferred crystalloid fluid for managing major surgery patients. The primary efficacy outcome was the proportion of patients with changes in renal function as assessed by creatinine concentrations during their index hospital admission. We used changes in creatinine to define acute kidney injury (AKI) according to the RIFLE criteria.*"

The results of the abstract now address feasibility end points, efficacy end pints and safety end points. We now state "*The study was feasible with 100% patient and clinician acceptance. There were no deviations from the trial protocol.*"

The results conclude with the statement "*There were no serious adverse events that related to the specific type of fluid received and there were no fluid crossover or contamination events.*"

The conclusion has been modified reflect feasibility, efficacy and safety. We now state "*The study design was feasible to support a future follow-up larger clinical trial. Patients treated with saline did not demonstrate an increased incidence of postoperative AKI (defined as changes in creatinine) compared to those treated with Plasma-Lyte. Our findings imply that clinicians can reasonably use either solution intraoperatively for adult patients undergoing major surgery.*"

Reviewer #3: Question 7. Patient population – please add to the limitations section the imbalance in the total number of patients in each study group, which is likely a product of the single crossover (but readers must be reassured this is not a product of post-enrolment exclusions preferentially in one group which would imbalance the study populations)
Authors' response, Question 7. Thank you for this excellent comment. In the limitations section we now state "*We recognise the inability of a single-cluster single-crossover design to address cluster and period effects. We did not analyse differences between the proportions of patients excluded from each cluster period. Neither did we undertake a sensitivity analysis of the primary outcome among all patients who received the blinded study fluid i.e., not limited to those who met post-enrolment eligibility criteria. We also recognise the imbalance in the total number of patients in each study group, which is likely a product of the single crossover design. Finally, we did not assess blinding i.e.,*

whether the clinicians using the fluid could correctly identify the whether the allocated fluid was saline or Plasma-Lyte. We acknowledge these factors as limitations of the study."

Reviewer #3: Question 8. Please clarify that patients undergoing a second surgery did not receive the study fluid during that surgery but they were still in the intention-to-treat population from their initial surgery, right? You did not throw out their initial enrolment, correct?
Authors' response, Question 8. This is an important comment, which we have addressed and clarifies in the methods section. We now state "*Patients requiring a second operation during the trial period who initially received the study fluid, still received the study fluid in their reoperation. They were included in the intention-to-treat population from their initial surgery, however excluded from the study for their reoperation."*

Reviewer #3: Question 9. Please add a statement to the discussion regarding why this clinical trial completed in February of 2015 is being made publicly available more than 5 years later. This happens in research, but funding agencies and other authorities are increasingly mandate release of RCT results within 12 months of completion – so some note of what difficulties were encountered that precluded this will help readers understand the context.
Authors' response, Question 9. Thank you for this question. Finally, whilst the trial was completed in 2015, the collection and checking of data was undertaken manually, crosschecked by two independent persons, and then reaudited, a process which took almost two years to complete. Additional delays can be attributed to the manuscript being rejected at other high impact journals and the coronavirus in 2020. Moreover, constructive review from reviewers at other journal also allowed us to address some of the limitation of the study and improve on the scientific merits of the study in its current submission to PLOS ONE. We do not think this delay impacts on the integrity or interpretation of our findings. We have briefly outlined this in the discussion section of the manuscript.

Reviewer #3: Question 10. Did you assess the success of the blinding? If yes, please present results. If not, please acknowledge in discussion as a limitation. (As you know, saline and balanced crystalloids have over effects on commonly measured labs and blinding is especially difficult to maintain in a cluster-level trial where all patients in the block are on the same fluid assignment)
Authors' response, Question 10. This is an excellent point that our research team were very conscious of for the duration of the study. Interestingly, we observed that clinicians using the trial fluid did not appear to make mention of what they thought the fluid was. In part, the cluster design resulted in consistency of the trial fluid being available at all times, and the fact that there was no available comparative crystalloid fluid to use was helpful. Importantly, when designing the labelling of the Trial fluids, we chose to name the Trial Fluid "Surgilyte" (see picture below, also included in the supplementary files), which also may have contributed to the high level of clinician acceptability. Initially, when designing this study, we thought it would be informative to formally survey clinicians using the fluid to assess how accurately they could identify the type of fluid used. We concluded that this may have had a negative impact with too much focus being projected on the type of fluid rather than maintain focus of the key study endpoints as we have outlined in the manuscript.

In the limitations section we state "*Finally, whilst the trial was completed in 2015, the collection and checking of data was undertaken manually, crosschecked by two independent persons, and then reaudited, a process which took almost two years to complete. Additional delays are attributed to the manuscript being rejected at other high impact journals and the coronavirus in 2020. We do not think this impacts on the integrity or interpretations of our findings."*

Blinded Plasma-Lyte

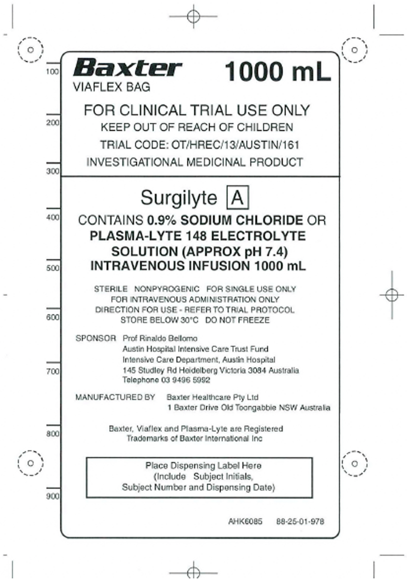

Blinded Saline

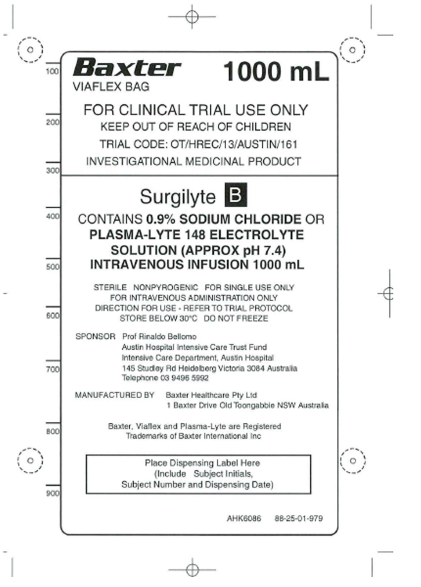

Reviewer #3: Question 11. Outcome assessment – please present more clearly in the results the number and proportion of patients in each group who did not have a creatinine measured with which to assess post-operative AKI (primary outcome) – and a P value to show that this proportion was the same between groups. This shows up in the discussion but I don't see it in the results.

Authors' response, Question 11. Thank you for this important comment. We have added in the following statement to the results section: *"Overall, there were 113 patients (10.6%) who were included in the trial who did not have their renal function measured as part of routine standard of care. The portion of patients in each group were similar (10.5% of patients receiving saline vs. 10.9% of patients receiving Plasma-Lyte, p = 0.841).*

Reviewer #3: Question 12. Please describe the process by which complications were graded by two independent clinicians

Authors' response, Question 12. We have now updated this in the Methods section. We now state "*Reporting and grading of complications were evaluated by two authors independently (MHL, CM) by undertaking an in-depth review of each patient's clinical records. In the case of disagreement, the case was presented to two other authors (LW, RB) to reach consensus."*

Reviewer #3: Question 13. In the discussion, "7942" should be "15802" when referring to the SMART trial.

Authors' response, Question 13. Thank you for picking up this important error. The SMART study enrolled 15,802 adults to receive saline or the balanced crystalloids (lactated Ringer's solution or Plasma-Lyte A). The 7942 patients that was stated in our original submission is incorrect. This number was the number of patients who received the balanced-crystalloids group. This has now been corrected.

Reviewer #4

We thank Professor JL Vincent for his valuable time taken to review our manuscript. Further, we appreciate his expert, constructive and considered review. Please find below a detailed response to each of the important comments.

Reviewer #4, Question 1. The authors compared the effects of saline to those of Plasmalyte in patients after cardiac surgery. In this double-blinded cluster, crossover trial, they enrolled 602 patients to the saline group and 458 to the Plasmalyte group. There was no major difference in biochemical findings. The study is somewhat old, but this should not influence the findings.
Authors' response, Question 1. Thank you for this question which has also been raised by Reviewer #3. Whilst the trial was completed in 2015, the collection and checking of data was undertaken manually, crosschecked by two independent persons, and then reaudited, a process which took almost two years to complete. Additional delays can be attributed to the manuscript being rejected at other high impact journals and the coronavirus in 2020. Moreover, constructive review from reviewers at other journal also allowed us to address some of the limitation of the study and improve on the scientific merits of the study in its current submission to PLOS ONE. We do not think this delay impacts on the integrity or interpretation of our findings. We have briefly outlined this in the discussion section of the manuscript.

Major comments

Reviewer #4, Question 2. The authors seem to ignore the basic mechanism by which saline solutions may alter the renal function, i.e., the hyperchloremia, with the secondary metabolic acidosis. Saline-treated patients had peak serum chloride levels at 103 [100 to 106] mmol/l vs 101 [98 to 103] mmol/L), there is no reason to believe in any difference in renal function. The conclusions of the present study are that the administration of 2 L of either saline or Plasmalyte does not affect the chloride levels and therefore cannot result in any difference in renal function, as assessed by the creatinine concentration. That is interesting.
Authors' response, Question 2. Thank you for this insightful question. In the introduction section of the manuscript, we state that controversy remains regarding the optimal fluid for use in this setting and that numerous studies in the past decade have examined the effects of hyperchloraemic solutions on renal function with most suggesting that balanced crystalloid solutions with lower chloride concentrations may have less nephrotoxic effects. We also now state in the introduction paragraph that "*Hyperchloraemia has been reported to be associated with chloride-induced renal vasoconstriction, however, more recent studies, have reported that major morbidity, including AKI, is comparable between patients treated with saline and those treated with lactated balanced crystalloids. Other studies have yielded conflicting results.*" These studies have also been referenced.

Reviewer #4, Question 3. The figure 2 is useless and could go in the supplement material. A figure showing the time course of chloride and bicarbonate is the essence of the paper.
Authors' response, Question 3. Given the comments made by Reviewer #3 (Question 1) pertaining to the Consort diagram, we respectfully think that the Consort diagram i.e., Figure 2 should remain in the manuscript. We have made significant modifications to Figure 2 that allows the reader to better understand the cluster crossover design used.

Reviewer #4, Question 4. Since AKI is assessed on the basis of creatinine concentrations and urine output, and the authors did not measure urine output, this is not AKI (I know some other papers did that, but this is not a reason to repeat the same mistakes). The authors should refer to a study of the renal function, as assessed by measurements of creatinine concentrations.
Authors' response, Question 4. This is an excellent comment, and we are very grateful for this suggestion. Accordingly, we have made numerous changes throughout the manuscript to reflect this change. This includes changes to the title, abstract, methods section, and discussion.

Reviewer #4, Question 5. Minor comment. The discussion section should briefly explain how the saline-induced hyperchloremia can influence renal function.
Authors' response, Question 5. Thank you for this excellent comment and suggestion. In the discussion we have now briefly outlined the proposed mechanism of hyperchloraemia and adverse renal function. We state that "*Numerous studies show that changes in serum chloride concentration, independent of serum sodium and bicarbonate, are associated with increased risk of AKI. Hyperchloraemia-associated AKI is thought to be related to renal vasoconstriction mediated by tubulo-glomerular feedback, and possibly other mechanisms, given the role of chloride in regulating renal blood flow, glomerular filtration rate, tubulo-glomerular feedback, and tubular injury.*"

Reference: Rein JL, Coca SG. "I don't get no respect": the role of chloride in acute kidney injury. Am J Physiol Renal Physiol. 2019 Mar 1;316(3):F587-F605. doi: 10.1152/ajprenal.00130.2018. Epub 2018 Dec 12. PMID: 30539650; PMCID: PMC6459301.

Once again, thank you for taking the time to review and consider our manuscript for publication in PLOS ONE. The comprehensive reviews and constructive comments provided by yourself and the expert reviewers have been immensely appreciated.

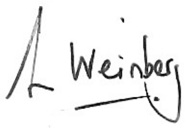

A/Prof Laurence Weinberg
BSc, MBBCh,MRCP,DPCritCareEcho,FANZCA,MD

Director, Department of Anaesthesia, Austin Hospital
Associate Professor, Department of Surgery, University of Melbourne
Associate Professor, Perioperative Pain and Medicine Unit, Department of Surgery, University of Melbourne
