# Supplementary material for: The effects of 0.9% saline versus Plasma-Lyte 148 on renal function as assessed by creatinine concentration in patients undergoing major surgery: A single-centre double-blinded cluster crossover trial
Source: PLoS One. 2021 May 19;16(5):e0251718. doi: 10.1371/journal.pone.0251718 (PMC8133498; doi:10.1371/journal.pone.0251718)
Supplement: Attachment — Submitted filename: RESPONSE TO REVIEWERS_PLOS_ONE_7_April_2021.pdf [file pone.0251718.s009.pdf]

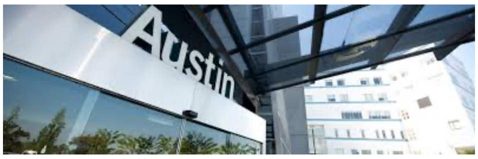

A/Prof Laurence Weinberg

Director
DEPARTMENT OF ANAESTHESIA
AUSTIN HEALTH

Tel: (03) 9496 3800 Fax: (03) 9459 6421
Email: Laurence.Weinberg@austin.org.au

Director of Anesthesia:
A/Prof Laurence Weinberg
BSc, MBBCh, MRCP,
FANZCA, MD

Admin Managers:
Mrs Val Hocking
Mrs Helen Pitman

Staff anaesthetists:
Dr Dishan Chandrasekara
MBBS, FANZCA
Dr Chong Tan
MBBS, FANZCA
Dr Louise Ellard
MBBS, FANZCA
Dr Brett Pearce
MBBS, FANZCA
Dr Raymond Hu
MBBS, FANZCA
Dr Audrey Bren
MBBS, FANZCA
Dr Lachlan Miles
MBBS, FANZCA
Dr Dean Cowie
MBBS, FANZCA
Dr Jonathan Fernandes
MBBS, FANZCA
Dr Jonathan Graham
MBBS, FANZCA
Dr Ian Harley
MBBS, FANZCA, FRCA
Dr William Howard
MBBS, Dip Med(Pain Mgt),
FANZCA, FFPMANZCA
Dr Antony Leaver
MBBS, FANZCA
Dr Frank Liskaser
MBBS, FANZCA
Dr Shiva Malekzadeh
MBBS, FANZCA
Dr Peter McCall
MBBS, FANZCA;
A/Prof Philip Peyton
MBBS, FANZCA, DA(UK),
DipObs(RACOG), MD
Dr Param Pillai
MBBS, MD Anes, FRCA
Dr Nick Scurrah
MBBS, FANZCA
Prof David Story
MBBS, FANZCA, MD
Dr David Tremewen
MBBS, FANZCA

RESPONSE TO REVIEWERS

PONE-D-20-37578R1
The effects of 0.9% saline versus Plasma-Lyte 148 on renal function as assessed by creatinine concentrations in patients undergoing major surgery: a single-centre double-blinded cluster crossover trial

On behalf of my co-authors, I would like to sincerely thank each Reviewer for considering our revised manuscript for publication. The comprehensive reviews and constructive comments provided by yourself and the expert reviewers have been immensely appreciated. The insightful and constructive comments have enabled us to strengthen the scientific rigor and merits of our research. Thank you once again for considering our resubmission for publication.

Reviewer 1: Professor Fernando Zampieri

Reviewer #1 All comments have been addressed. I have no further comments. I commend the authors for their work.

Authors' reply. Thank you once again for taking the time to review our resubmission manuscript and for your positive comments.

Reviewer 2: Dr Sanjeev Sarmukaddam

Reviewer 2: All the comments made on earlier drafts by me (and hopefully by other respected reviewers also) were/are attended positively/adequately, I am fully satisfied, and the manuscript is improved a lot. Excellent job. I recommend acceptance. Quoted article on 'Understanding the cluster randomised crossover design: a graphical illustration of the components of variation and a sample size tutorial' is excellent and new for me. Thanks a lot.
Authors' reply. Thank you Dr Sarmukaddam for your expert comments and for taking the time to review our revised manuscript.

Reviewer 4: Professor JL Vincent

Reviewer 4: The authors do not seem to understand the basic concepts. They fail to recognize the basic mechanism by which saline solutions may alter the renal function, i.e., the hyperchloremia, with the secondary metabolic acidosis. Saline-treated patients had peak serum chloride levels at 103 [100 to 106] mmol/l vs 101 [98 to 103] mmol/L), there is no reason to believe in any difference in renal function. The conclusions of the present study are that the administration of 2 L of either saline or Plasmalyte does not affect the chloride levels and therefore cannot result in any difference in renal function, as assessed by the creatinine concentration. That is interesting.

In the study by Stemler et al (N Engl J Med 2018), there were more MAKE in the saline treated patients, but their chloride levels increased to a mean of 112 mmol/L. For explanations, to can refer to Vincent and DeBacker 'we do not appreciate SALT' in the Am J Resp Crit Care Med 2017.

A figure showing chloride levels over time is missing, when this is the core of the paper. This information should be included in the conclusions of the paper. Even pragmatic clinical trials must include a sound scientific rationale.

Question 1. Authors' response: Thank you once again for your constructive comments. Once again, we feel privileged to have such an internationally distinguished academic clinician review our manuscript. We are genuinely grateful for your advice regarding further modifications to our manuscript.

We are very familiar with the excellent paper by Semler and we have already made reference to this in our manuscript. The article by Vincent JL, De Backer D. We Do Not Appreciate SALT. Am J Respir Crit Care Med. 2018 May 15;197(10):1361 is also well known to our group. We acknowledge many of the important issues regarding hyperchloraemia that were raised in this thought-provoking Editorial. We agree with Professor Vincent, as stated in the Editorial, that further information discussing the potential harmful effects of hyperchloraemic solutions would strengthen our manuscript.

We have now included the following addition information in the discussion section of the revised manuscript. We have also included four more references supporting our discussion.

In the third paragraph of our discussion, we state now state *"Our findings imply that crossover design studies investigating fluid therapy in the perioperative setting are feasible. Moreover, our findings suggest that clinicians can reasonably use either solution intraoperatively for adult patients undergoing major emergency or elective procedures if the volume of fluid administered is less than 2000 mL. However, concerns about the potentially harmful effects of hyperchloraemia remain. The clinical practice of using IV fluid solutions with unphysiological concentrations of chloride has been strongly discouraged due to the significant association between hyperchloraemia and the development of metabolic acidosis and adverse renal outcomes [30-32]. Moreover, for clinical trials investigating the effects of hyperchloraemia versus balanced solutions,*

clinical equipoise has also been recently questioned for the continued use of hyperchloremic solutions. This is particularly true if patients who develop hyperchloraemia continue to receive solutions with supraphysiological concentrations of chloride [30]. Given that numerous studies have shown a harm signal associated with hyperchloraemic crystalloid solutions, without a comparable benefit signal [33], the ethical integrity of clinical trials comparing saline to balanced solutions has also come under scrutiny [30].

In the present study, we acknowledge that the low chloride load associated with the administration of small volumes of saline was well tolerated by our patient cohort, who were at low risk for AKI. Moreover, we also acknowledge that our findings are not generalisable to patients with pre-existing renal dysfunction, those with metabolic acidosis and those receiving large volumes of hyperchloraemic solutions [31]. Recent editorials have emphasised that renal function is frequently unknown during surgery, and the volume of fluid replacement is unpredictable in acute care settings [31]. Therefore, because there is no evidence of benefit from hyperchloraemic solutions (other than in the management of metabolic alkalosis or hyponatraemia), and there is evidence of potential harm, there appears to be no physiologic or clinical justification for the continued use of "unphysiological" saline (or any other unbalanced crystalloid solution) for perioperative volume therapy, especially when safer and more physiological solutions are available [31,32,33].

Additional references

30. Vincent JL, De Backer D. We Do Not Appreciate SALT. Am J Respir Crit Care Med. 2018 May 15;197(10):1361. DOI: 10.1164/rccm.201709-1874LE.
31. Priebe HJ. Another nail in the saline coffin. Br J Anaesth. 2018 Jun;120(6):1432-1434. DOI: 10.1016/j.bja.2018.02.026.
32. Kellum JA, Shaw AD. Assessing toxicity of intravenous crystalloids in critically ill patients. JAMA 2015 Oct 27;314(16):1695-7. DOI: 10.1001/jama.2015.12390.
33. McLean DJ, Shaw AD. Intravenous fluids: effects on renal outcomes. Br J Anaesth. 2018 Feb;120(2):397-402. DOI: 10.1016/j.bja.2017.11.090.

Question 2. Authors' response: In addition, we have now included another Figure showing chloride levels over time. The addition of Figure 3 graphically outlines the preoperative and postoperative changes in chloride levels. We have provided violin plots rather than the traditional box plots. We think that violin plots are more informative than a plain box plot, which only shows summary statistics such as median and interquartile ranges. In contrast, the violin plot shows the full distribution of the data. The difference is particularly useful when the data distribution is multimodal (more than one peak). By using violin plots, we hope to demonstrate the presence of different chloride peaks, their position and relative frequency.

Figure 3. Preoperative and postoperative changes in chloride levels. The single thicker dashed line represents the median value, and the thinner dashed lines represent the interquartile ranges.

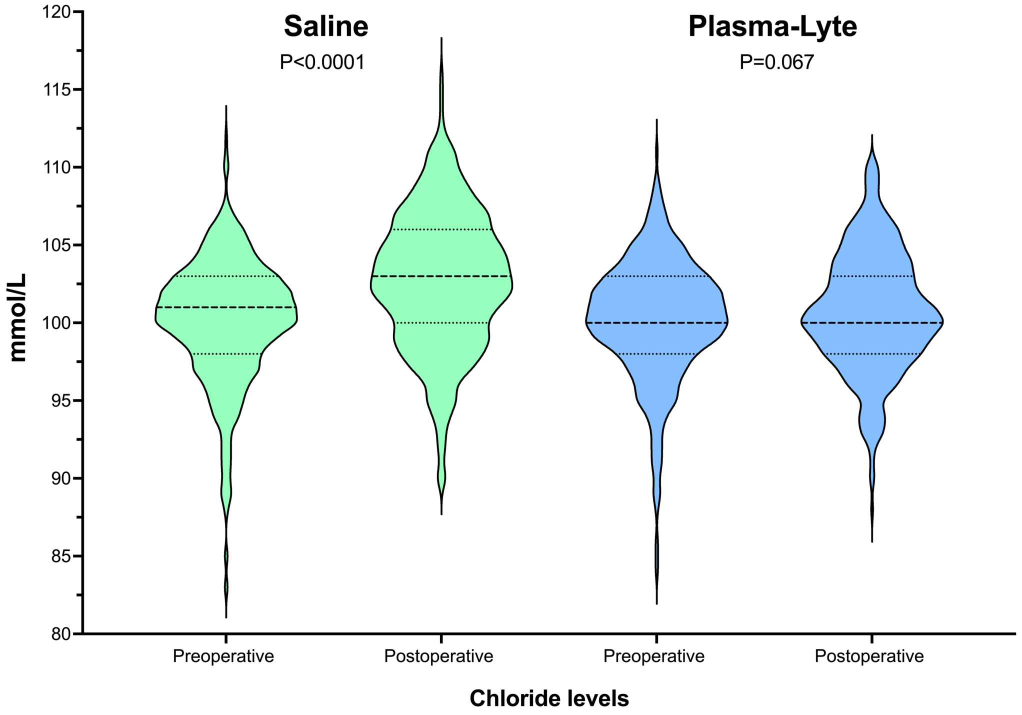

Question 3. We have also deleted the sentence in the conclusion stating that *"Our findings also imply that clinicians can reasonably use either solution intraoperatively for adult patients undergoing major surgery."*

In summary, we hope we have adequately addressed the constructive comments by Professor Vincent and would be very happy to make further modifications if requested. Once again, we thank Professor Vincent for taking the time to review and consider our manuscript for publication in PLOS ONE. His comprehensive review and constructive comments provided, in addition to the positive comments from the other reviewers during the first resubmission have been immensely appreciated.

Sincerely,

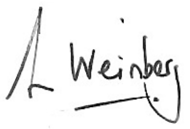

A/Prof Laurence Weinberg
BSc, MBBCh,MRCP,DPCritCareEcho,FANZCA,MD

Director, Department of Anaesthesia, Austin Hospital
Associate Professor, Department of Surgery, University of Melbourne
Associate Professor, Perioperative Pain and Medicine Unit, Department of Surgery, University of Melbourne
