# Supplementary material for: The effects of 0.9% saline versus Plasma-Lyte 148 on renal function as assessed by creatinine concentration in patients undergoing major surgery: A single-centre double-blinded cluster crossover trial
Source: PLoS One. 2021 May 19;16(5):e0251718. doi: 10.1371/journal.pone.0251718 (PMC8133498; doi:10.1371/journal.pone.0251718)
Supplement: Attachment — Submitted filename: renamed_b85c2.docx [file pone.0251718.s007.docx]

**COMMENTS – Manuscript PONE-D-20-37578**

Title: “The effects of 0.9% saline versus Plasma-Lyte 148 on acute kidney injury in patients undergoing major surgery: a single-centre double-blinded cluster crossover trial”

**Important note: This review pertains only to ‘statistical aspects’ of the study and so** **‘****clinical aspects’ [like medical importance,** **relevance of the study,** **‘clinical significance and** **implication(s)’ of the whole study, etc.] are to be evaluated [should be assessed] separately/independently. Further please note that any ‘statistical review’ is generally done under the assumption that (such) study specific methodological [as well as execution] issues are perfectly taken care of by the investigator(s). This review is not an exception to that and so does not cover clinical aspects {however, seldom comments are made only if those issues are intimately / scientifically related & intermingle with ‘statistical aspects’ of the study}. Agreed that ‘statistical methods’ are used as just tools here, however, they are vital part of methodology [and so should be given due importance].**

**COMMENTS:** Actually, your ABSTRACT is well drafted but assay type [though you started with ‘Background’, I guess with intention to divide the ABSTRACT in sections]. Please note that it is preferable [refer to item 1b of CONSORT checklist 2010: Structured summary of trial design, methods, results, and conclusions] to divide the ABSTRACT with small sections like ‘Objective(s)’, ‘Methods’, ‘Results’, ‘Conclusions’, etc. which is an accepted practice of most of the good/standard journals [including this one]. It will definitely be more informative then, I think, whatever the article type may be.

I very strongly feel (and so have a very serious objection) that inclusion of the term ‘**crossover trial**’ is by [I guess] oversight/mistake. Note that the cross-over design {as per my knowledge of ‘standard definition’ and ‘ideal/original concept’} is a special case of a randomized control trial that allows each subject to serve as his own control. Following is a quote from a standard text-book:

In order to use the cross–over design, assumption to be made is that the effect of the intervention during the first period must not carry–over into the second period (carry–over effect or spill-over effect). Therefore, it is necessary that the therapies under study have no carry–over effect. Differential carry-over effect may be eliminated by interposing a long dry–out or wash–out period (the length of which is determined by the pharmacological properties of the drugs being tested) between the termination of the treatment given first and the beginning of the treatment given second.

I am sure that the authors know above quoted things. However, please specify clearly if you intended to imply something else (some other concept) by the term ‘**crossover trial**’ [remember that this is a scientific/academic document and so all details should be clearly/correctly communicated]. In fact, use of (standard) crossover trial in the field of ‘surgery’ is rare and so is highly appreciated [if it is correctly adapted/used]. Further read the following paragraph from the same book:

The appeal of this design is to avoid ‘between – subject’ variation in estimating the intervention effect, thereby all patients can be assured that sometime during the course of investigation, they will receive the new therapy {both treatments}. However, this method of study is not suitable if the drug of interest cures the disease, if the drug is effective only during a certain stage of the disease or if the disease changes radically during the period of time required for the study.

In table-2, you compare two treatment [Plasma-Lyte vs Saline after surgery] with different sample (group) sizes [in real crossover trial the group sizes are same as each subject will receive both treatments ultimately (only in different ‘order’)]. One known variation is ‘case-crossover’ design. This design is useful when the risk factor/exposure is transient. Data yielded by this design are analyzed as of ‘matched case-control’ studies. I do not see any of these [concept used as per standard] anywhere in this manuscript.

All the methodologically important concepts [like double-blinding, clustering, crossover trial] adapted/used here are not made clear. Account given {example regarding double-blinding described in lines 104-118 & also figure-1 regarding ‘Cluster crossover design of Saline vs Plasma-Lyte study’ does not depict clearly the ‘crossover’ nature, it mainly gives ‘time line’} is ‘confusing’, I feel. Advantages generally known of double-blinding are applicable for “parallel independent groups” design. For ‘crossover’ design (except avoidance of ‘auto- and hetero-suggestion’ bias) they may differ, I guess.

Moreover, all ‘statistical analyses’ performed here [example, line 164-65: Comparisons between categorical variables were made using chi-square and Fisher’s exact tests, while continuous variables were compared using the Mann–Whitney U test. including estimation of IRR, HR, all 95%CIs] assume ‘independence of the groups/samples’ which is violated in case ‘crossover trial’.

In my opinion, it [the ethics committee granting waiver of participant consent] is not a standard (practice) for any reason [like given in lines 77-79: participation in the research posed no more than minimal increase in risk to individuals than what they would be exposed to if they were not in the study, the ethics committee granted waiver of participant consent]. Other analyses {as described in lines 172-180 (We conducted time-to-event analyses using Cox proportional hazard regression models, and compared non-dichotomous outcomes using Poisson or negative binomial regression models, all adjusted for the same set of covariates, with treatment effects reported as adjusted hazard ratios with 95% CIs. The threshold for statistical significance was a P value of 0.05. All secondary and exploratory endpoints are reported as point estimates of treatment effects with 95% CIs.^29^ We report both covariate-adjusted and unadjusted outcomes. We used forest plots to present the primary and key secondary outcomes regarding the consistency of a treatment effect across the subgroups)} are alright.

Fig 2. CONSORT diagram and Fig 3. Incidence rate ratios of key outcomes for patients receiving Plasma-Lyte compared to Saline are also good. Overall, this article needs to be re-drafted and also change in title considering above highlighted points, I guess.
